# DAIKON: A Data Acquisition, Integration, and Knowledge Capture Web Application for Target-Based Drug Discovery

**DOI:** 10.1021/acsptsci.3c00034

**Published:** 2023-06-22

**Authors:** Siddhant Rath, Saswati Panda, James C. Sacchettini, Steven J. Berthel

**Affiliations:** † Department of Biochemistry & Biophysics, Texas A&M University, College Station, Texas 77843, United States; ‡ Panorama Global, Seattle, Washington 98121, United States

**Keywords:** drug discovery, target based, database, knowledge-capture, software, data repository, portfolio management

## Abstract

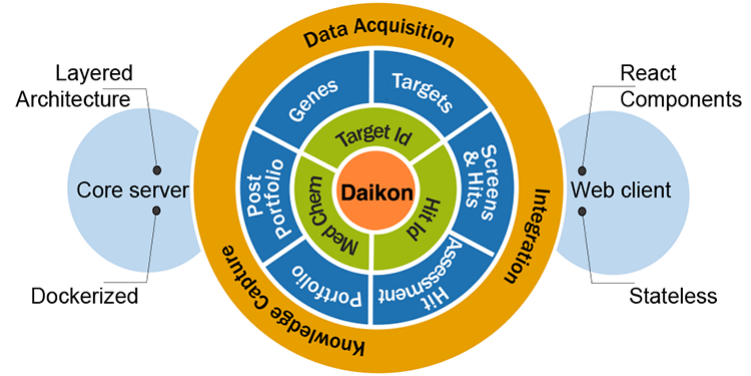

Primitive data organization practices struggle to deliver at the scale and consistency required to meet multidisciplinary collaborations in drug discovery. For effective data sharing and coordination, a unified platform that can collect and analyze scientific information is essential. We present DAIKON, an open-source framework that integrates targets, screens, hits, and manages projects within a target-based drug discovery portfolio. Its knowledge capture components enable teams to record subsequent molecules as their properties improve, facilitate team collaboration through discussion threads, and include modules that visually illustrate the progress of each target as it advances through the pipeline. It serves as a repository for scientists sourcing data from Mycobrowser, UniProt, PDB. The goal is to globalize several variations of the drug-discovery program without compromising local aspects of specific workflows. DAIKON is modularized by abstracting the database and creating separate layers for entities, business logic, infrastructure, APIs, and frontend, with each tier allowing for extensions. Using Docker, the framework is packaged into two solutions: daikon-server-core and daikon-client. Organizations may deploy the project to on-premises servers or VPC. Active-Directory/SSO is supported for user administration. End users can access the application with a web browser. Currently, DAIKON is implemented in the TB Drug Accelerator program (TBDA).

In recent decades, the volume of data collected and evaluated during drug discovery programs has risen, as has collaboration between academic and pharmaceutical research groups,^1^ which is essential to contemporary drug discovery strategies. During the course of a typical ″target-based″ approach, multiple processes, such as target identification and validation, high throughput screening, hit identification, hit-to-lead (H2L), etc., are routinely employed, leading to the generation of a variety of data.^2^ Information flows continuously within an ecosystem of interrelated individuals and teams from academia, project managers, non-profit organizations, and the pharmaceutical and biotech industries.^3^

In a typical academic research laboratory setup, experimental findings are saved locally, in network drives using spreadsheets, or communicated through email, adhering to rudimentary methods. This presents a difficult challenge as data are frequently siloed and unable to flow freely across the community’s network of collaborators, which causes duplication of work, loss of valuable time and effort by scientists, and hinders open innovation.^4^ Aside from the uncertainties inherent in scientific research, one of the other challenges that occur in this process is managing multiple complicated portfolios, including several projects at various phases of drug discovery. Most projects in the portfolio or post-portfolio stage in a multidisciplinary network are scattered and distributed in the form of reports, smart sheets, or other external databases across numerous independent tools that may or may not be interconnected. As a result, there is an apparent discontinuity in the workflow, which diminishes the overall efficiency of the team. Scientists can be extremely successful in developing breakthrough insights about hit compounds within the confines of their own sphere; however, without productive collaborations and integrations with other domains of the consortium, such knowledge may be largely unusable to the advancement of drug discovery.

Researchers have access to a variety of publicly accessible biological and chemical databases, such as Mycobrowser,^5^ PDB,^6^ChEMBL,^7^ etc., but these are not expected to permit users to maintain their information management. Popular project management tools like Smartsheet, JIRA, etc. can manage project portfolios and facilitate team collaboration. Nevertheless, utilizing advanced biochemical features within these platforms, such as integrating chemical databases, generating a structure from a SMILES string within a project, visualizing 3D structures, and generating a one-to-one connection between drug targets and projects, has proven difficult. Several pharmaceutical corporations build in-house tools with higher customizability to address these difficulties that are often unavailable to the academic community. Other commercial systems, such as ChemAxon,^8^ JChem,^9^ etc., provide modular solutions that address some of these challenges but bear a licensing cost. During our research, we discovered proprietary tools that are currently being utilized in pharma; however, we could not find open-source solutions in the literature.^10^ The need for such a solution that can enable research groups to effectively collaborate, as well as assist program and portfolio administrators in managing their projects, is unmatched. Visualizing a compound’s journey from a potential target to a clinical candidate would benefit the scientific community.

We introduce an open-source solution—DAIKON (Data Acquisition, Integration, and Knowledge capture application)—which bridges the gaps between the scientific and project management components of interdisciplinary collaboration. The framework aims to address challenges caused by using separate disconnected applications, the lack of functionalities to effectively capture scientific results, and the absence of a start-to-end tracking interface that associates scientific research data from targets with projects and portfolios.

This paper is structured as follows: First, we outline the design, covering the application’s primary features, as they correspond to the drug discovery workflow. The technical architecture and implementation are described in the next section, followed by a use case for the TBDA.^11^ Finally, we explore the application’s future directions.

## Overview

A target encompasses a variety of biological entities, such as proteins, genes, and RNA, which are associated with disease pathogenesis. In a target-based approach, selecting a target as the pivot provides advantages over other strategies since it leads to projects and portfolios based on compounds connected to a particular or a set of targets.^12^ In accordance with this methodology, therefore, we identify “targets” as the primary key that binds the application together in contrast to chemical databases and project management solutions that are centered on “compounds” or “projects”.

**Figure 1:**
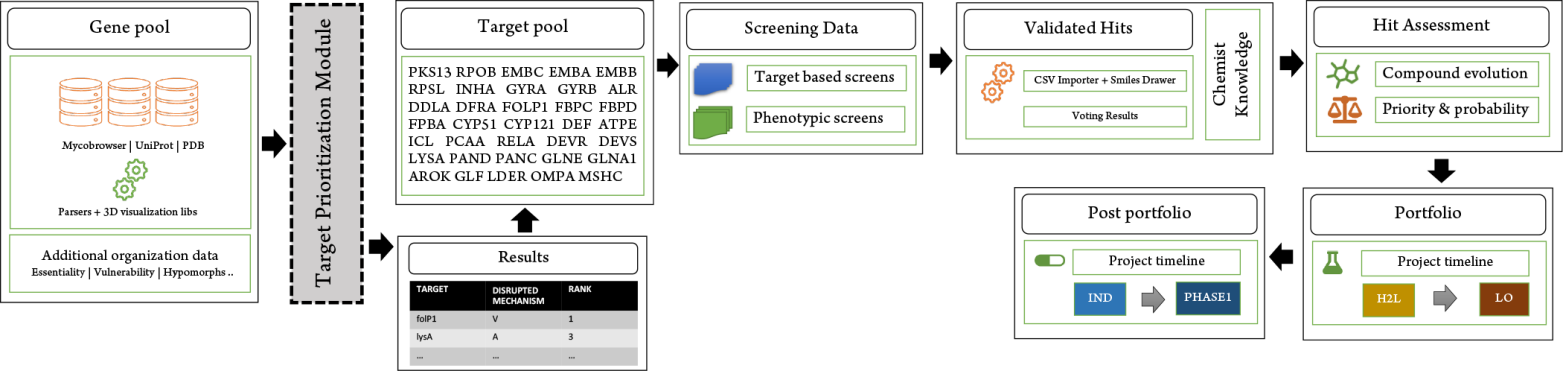
Typical TB drug discovery workflow in DAIKON.

One of the most important components of drug discovery is the target selection procedure, which involves sourcing data from publications and public databases like Mycobrowser and PDB, among others. DAIKON acquires this information and includes additional fields like essentiality, vulnerability, protein production lists, etc., which can be modified and recorded by the scientific research group. This information is paired with the analysis of a target prioritization module to choose a potential target. In addition to the screening techniques, a list of screening efforts conducted on the target is captured. To enable medicinal chemists to select qualifying hits as a starting point for hit evaluation, the application incorporates a voting system. The hit assessment stage entails more research on the voted hits in order to enhance the structures. These enhancements are versioned in a ″compound evolution″ component to monitor structural modifications and molecular characteristics, forming a historical timeline. This stage also signifies the formation of a project that will allow the compound to advance to the portfolio and post-portfolio stages further along the pipeline. The application keeps track of project timeframes, planned/estimated dates, status, and priority, as well as any project terminations. These processes are completed in collaboration with several organizations and can take a considerable amount of time. Each target and its phases are labeled with a discussion thread functionality, increasing user involvement. The application blends the journey of a target through its numerous screening phases with a range of validated hits, projects, and portfolios into a single holistic tree-like structure termed “horizon view,” which may be spawned for a target in any of its stages. DAIKON’s typical workflow for discovering TB drugs is depicted in Figure 1.

Technically, the architecture has been heavily modularized to increase the solution’s life expectancy, keeping in mind the prolonged nature of drug discovery projects and attempting to overcome roadblocks posed by expiring third-party dependencies by enabling targeted upgrades of specific affected modules. We adhered to sustainable and maintainable design patterns that can rapidly adjust to design and policy changes in the drug discovery pipeline, organization-specific adaptations, and other variations with relative ease.

## Design

The tool organizes the discovery pipeline using an “orthogonal approach”, positioning Gene, Target, Screen, Hit Assessment, Portfolio, and Post-Portfolio on the horizontal axis, while introducing a vertical arrangement of information, beginning with a high-level overview and progressing down to more specific data, to adjust the amount of granularity presented at each stage of the process.

**Figure 2:**
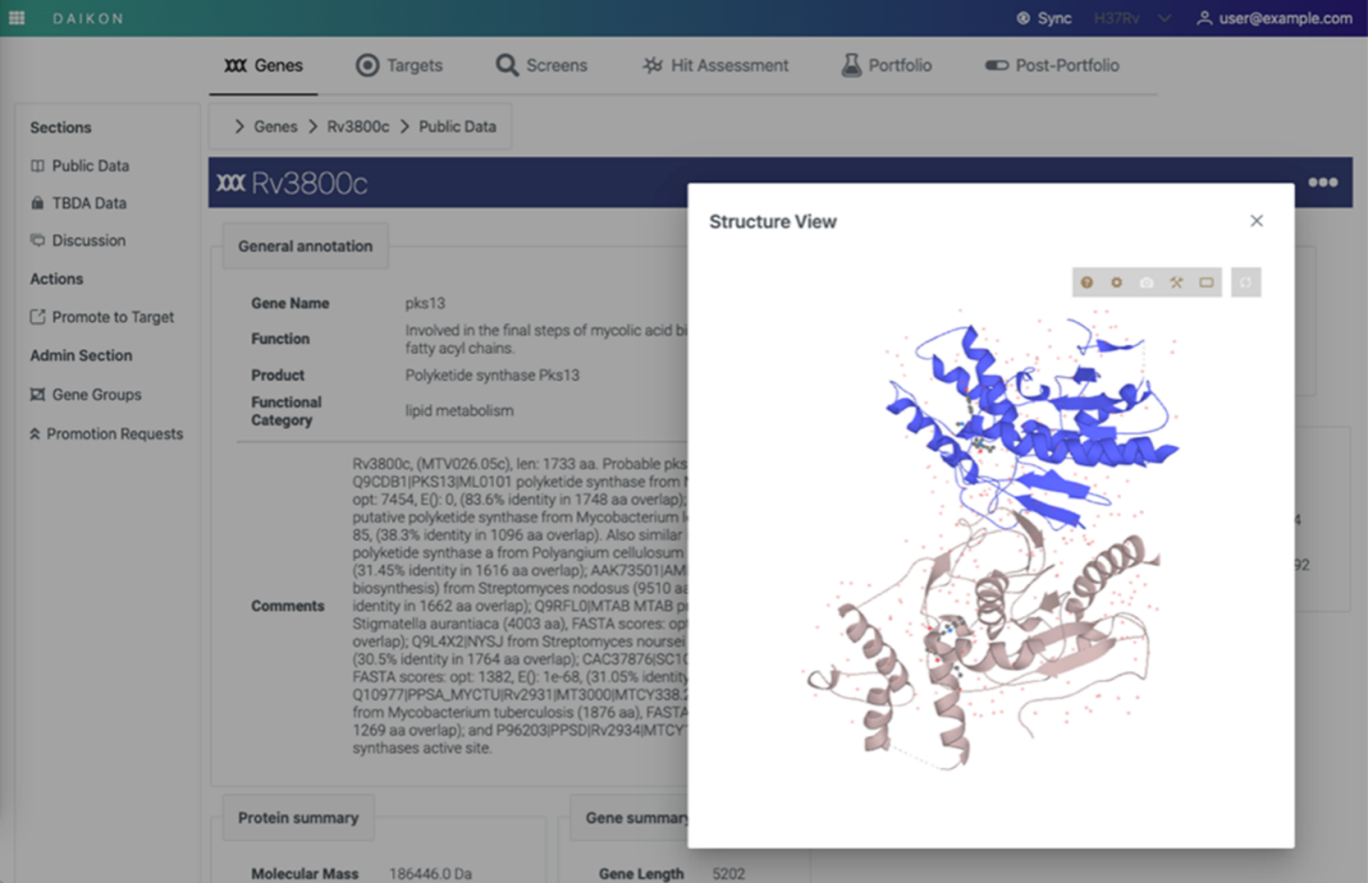
Crystal structure of the Mtb Pks13 thioesterase domain in complex with inhibitor TAM16 in embedded structure viewer.^15^

As a starting point for the horizontal axis, we use the Gene component containing approximately 4173 genes from the most used laboratory strain of *Mycobacterium tuberculosis* (Mtb) H37Rv sourced from Mycobrowser. The application provides a tabular overview of genomic sequence, orthologues, coordinates, as well as protein summary information and protein sequence. Additionally, structures, chains, and ligands are retrieved in real time from PDB and UniProt.^13^ DAIKON incorporates the LiteMol suite,^14^ an interactive and responsive structure viewer that facilitates 3D visualization of these structures, as shown in Figure 2.

In addition, the app collects data exclusive to member organizations, such as essentiality, vulnerability, protein production, hypomorphs, CRISPRi-based gene information, etc. According to the organization’s regulations, all members or a specified group may enter these data. DAIKON has integrated several features and functionalities to facilitate effective documentation of data provenance by users. Specifically, users can annotate their data with essential metadata, which is captured and stored in fields such as URLs, DOIs, references, and notes, providing comprehensive documentation of the data sources. Any modifications are automatically versioned and may be monitored using the “version history” feature. The versioning schema is based on a combination of the GUID of the entity, name, date time stamp, and the editor’s user id. Versioning is done at a granular level, where each data attribute is versioned individually, rather than the dataset as a whole. When an attribute is created, modified, or deleted, a tracking entry is generated, which includes important details such as the entity name, property name, old and new values, user information, and modification date and time. These entries are stored in an attribute-tracking-log table to create a “version history feature” in the GUI, allowing users to view changes in a timeline format.

Using the “target prioritization module,” the protein associated with a certain gene or set of genes (protein complex) may be promoted as a possible therapeutic target from the gene. In general, this module uses quantitative or qualitative domain-specific approaches to define targets based on specified criteria.^16,17^ For example, prioritization of cancer therapeutic targets can be performed using CRISPR-CAS9 screens.^18^ Similarly, for coronary artery disease, causal genes are prioritized based on experimental and in silico evidence using the SMR/HEIDI strategy.^19^ In TB, parameters such as druggability and in vitro essentiality can be utilized to evaluate and rank targets.^20^ Given that there are various prioritizing approaches,^21^ this module is represented in the framework as an abstract form and must be implemented based on the research team’s chosen methodology. The output of the prioritizing module is stored in the application, and the protein advances to the “target component” depending on the evaluation findings. This component includes a hierarchy of information views about the feasibility of promoted targets. A tabular overview includes a list of all targets, their associated genes, and evaluation criteria utilized by the research team. In addition, graphs and charts can be incorporated into this component to further illustrate the target priority landscape, provided the prioritization module offers the necessary parameters. For each target of interest, a scorecard is created that includes a detailed perspective and is conditionally color-formatted depending on a logic set by the project managers that results in the target’s ranking. Compass view is a second summary view provided by the component. It gives the most condensed version of target information and consists of four quadrants: Background, Enablement, Strategy, and Challenges. Several computations and responses from the previous module inform the majority of these sections.

The targets are then continuously screened using a variety of techniques such as high-throughput screening,^22^ phenotypic,^23^ DNA-encoded,^24^ and virtual screening.^25^ While the major purpose is to capture knowledge from target-based strategies, we also allow for phenotypic screening data, which is often used as a first step prior to the identity of a specific drug target. The app has been adapted to accommodate target-agnostic situations. As such, it is flexible enough to handle situations that serve both approaches. DAIKON captures data from the screening efforts in the screen component by recording information such as screening methodology, organizations involved, protocols, inhibitor concentration, the number of compounds tested, and start and end dates.

The “Validated hits” section presents a summary of quality hits, together with additional information such as enzyme activity (IC50), MIC, structural data, library/source, etc. that have been confirmed as hits during the screening phase. Typically, chemists evaluate this list largely based on factors such as molecular weight, cLogP, and polar surface area to determine the most promising hits.^26^ We handle this selection process with a voting mechanism that allows them to rate these compounds and prioritize the high-quality hits to proceed to the next phase. At this point, the team will assess the profile of the rated hits and advance them to the Hit Assessment (HA) stage.

**Figure 3:**
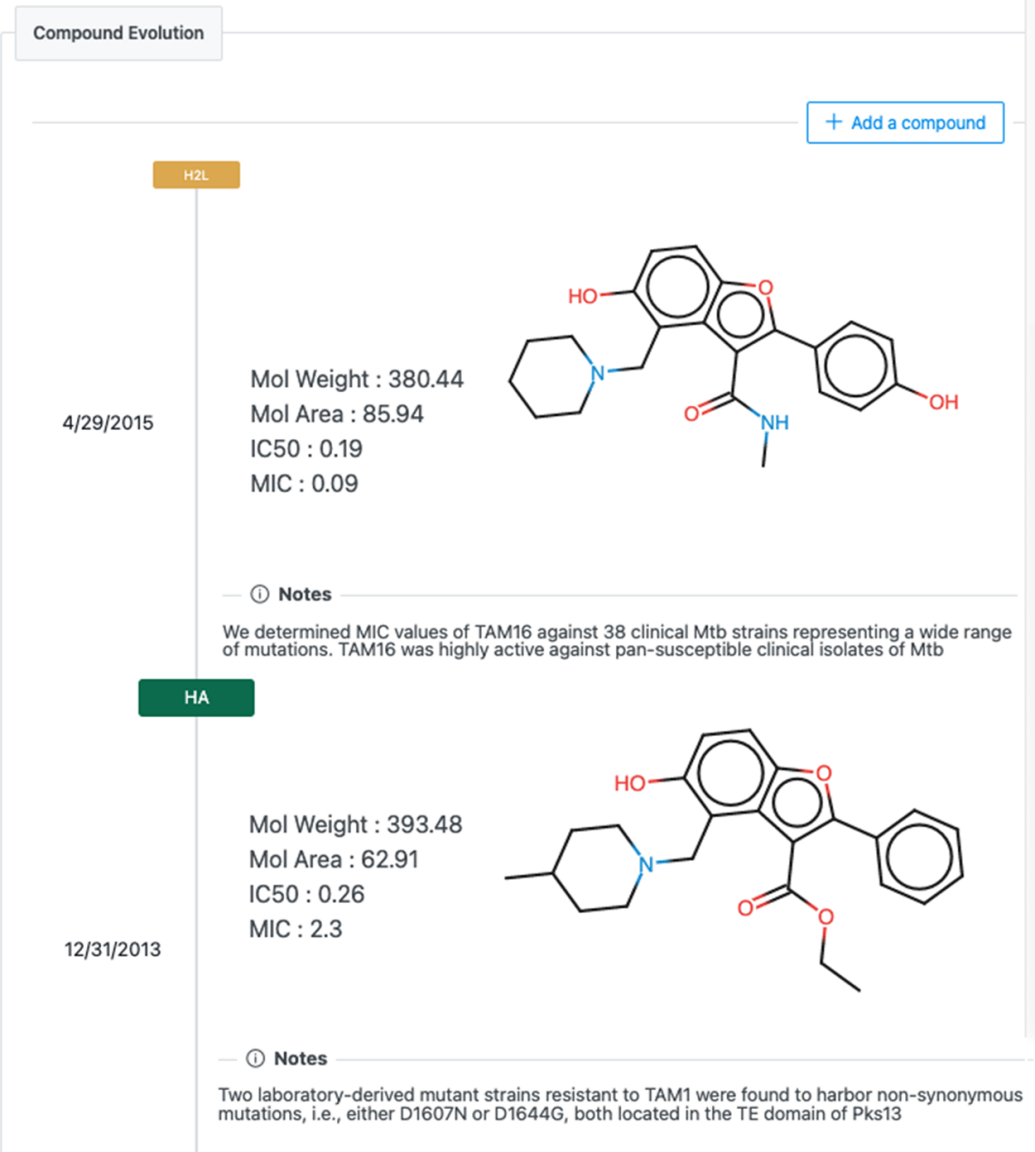
Structure-Guided Development of TAM16 in “HA” and “H2L” stages as captured in the Compound Evolution Component.

The HA component offers a comprehensive view of all ongoing projects filtered by target, displaying pertinent information including HA status, milestone dates, chemical structures, and selected biological or physicochemical attributes of compounds. Additionally, the component presents a status indicator for both active and terminated projects, allowing for efficient tracking of project progress. DAIKON features a knowledge capture component, “Compound Evolution,” which enables the project team to record details of successive molecules as properties are improved. By preserving snapshots of each molecule, including the initial hits, it is feasible to obtain a comprehensive overview of the series. This enables users to not only assess the progress of a project but, in retrospect, learn by example how issues and challenges were addressed and, in some cases, overcome. Figure 3 shows the structural development of TAM16 (Pks13) in the compound evolution module. In addition, as an indication of the level of resources on a project, DAIKON allows for a team to set the project’s relative or absolute priority. Finally, the team, or a reviewer, can also assign a probability assessment to a project. Priority and probability assessments on multiple active projects can also allow review boards and portfolio managers to monitor the overall health and potential of a portfolio of projects.

If a hit series is assessed to be viable, a formal decision to admit it to a program’s portfolio is made and the project advances to the next stage. The H2L stage, in many organizations or consortiums, represents the point at which a full medicinal chemistry team is committed to the project. During this stage, structure activity and property relationships are further established and solutions to factors such as in vitro biological activity (enzymatic or cellular), absorption, distribution, metabolism, excretion, or in vitro toxicity are sought.^27^ If satisfactory molecules are identified and a certain degree of biological activity is established, typically in an in vivo disease model, a lead series is declared, and the project can advance to the lead optimization stage.^28^ At this point, medicinal chemistry resourcing may increase and is focused on further addressing outstanding issues and improving in vivo activity. Pharmacokinetics, pharmacodynamics, more sophisticated in vitro and in vivo toxicity assays, and studies may all be explored in order to identify a compound or small set of compounds that may be suitable for clinical candidate selection.^29^ Prior to candidate nomination, additional stages may be required depending on the disease, therapy area, or organization. In all cases, criteria for advancement and informal/formal processes will be conducted external to DAIKON. Administrative features within DAIKON allow for easy stage transition or termination when these types of decisions are made. Although DAIKON was originally conceived as a tool to allow for project tracking and knowledge capture in the pharma discovery phase (i.e., up to candidate selection), provisions for following candidates through early development have been made. In the post-portfolio component, the familiar structure of the portfolio view is preserved, with the inclusion of milestone dates corresponding to the filing of an investigational new drug and the commencement of first-in-human (phase 1) studies. Considering the complexities of clinical development and the likelihood that further advancement will involve partner organizations with data systems that are specially designed to accommodate the data and regulatory demands in this phase, no effort has been made to extend DAIKON’s scope further into this area.

**Figure 4:**
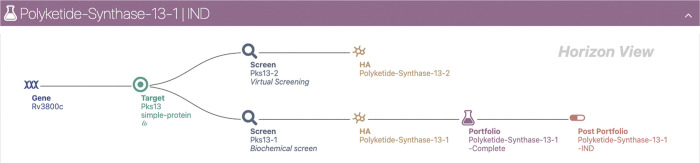
“Horizontal View” showcasing an example of the progression of target Pks13 in the pipeline, highlighting two projects that originated from two distinct screens.

In addition to the previously described components that capture individual drug discovery data points, DAIKON also provides a visual representation of the progress for each target and project through the “Horizon View,” which is applicable to all stages of the pipeline. This view is depicted as a horizontal tree with the selected gene serving as the root, and each branch signifies the progress at different stages, enabling an intuitive understanding of a gene’s journey from start to finish, as shown in Figure 4.

A discussion thread is another DAIKON feature that encourages continual communication and interaction among members. It is connected to a target and tagged at every stage, with each chain of thought carried forward as the stage advances.

As stated previously, DAIKON allows for a ″dual focus″ of information. Along with the horizontal progression of information from gene to candidate selection, there is a vertical arrangement of information from high-level overview to more detailed project data. In this way, different types of users can access information at a level that is most useful for them. For project and portfolio managers seeking information on the entire program of projects, or projects that address a common target or mechanism of action, the high-level views showing rolled-up data are most useful. For scientists working on the same project or target, more detailed information and the ability to comment on results or approaches is advantageous. Regardless of the role, moving vertically or horizontally within the application is easy and should facilitate understanding of a project or program of projects.

## Technical Architecture and Implementation

DAIKON is developed using industry-standard frameworks in .NET core and React JS in a client–server architecture that communicates using JSON APIs. The framework is intended to be deployed for a multidisciplinary collaboration having access to either an on-premise server or a private cloud. The client is a web-based application compatible with all modern web browsers that support HTML5 and JavaScript. No installation on the user’s system is required.

### Infrastructure

**Figure 5:**
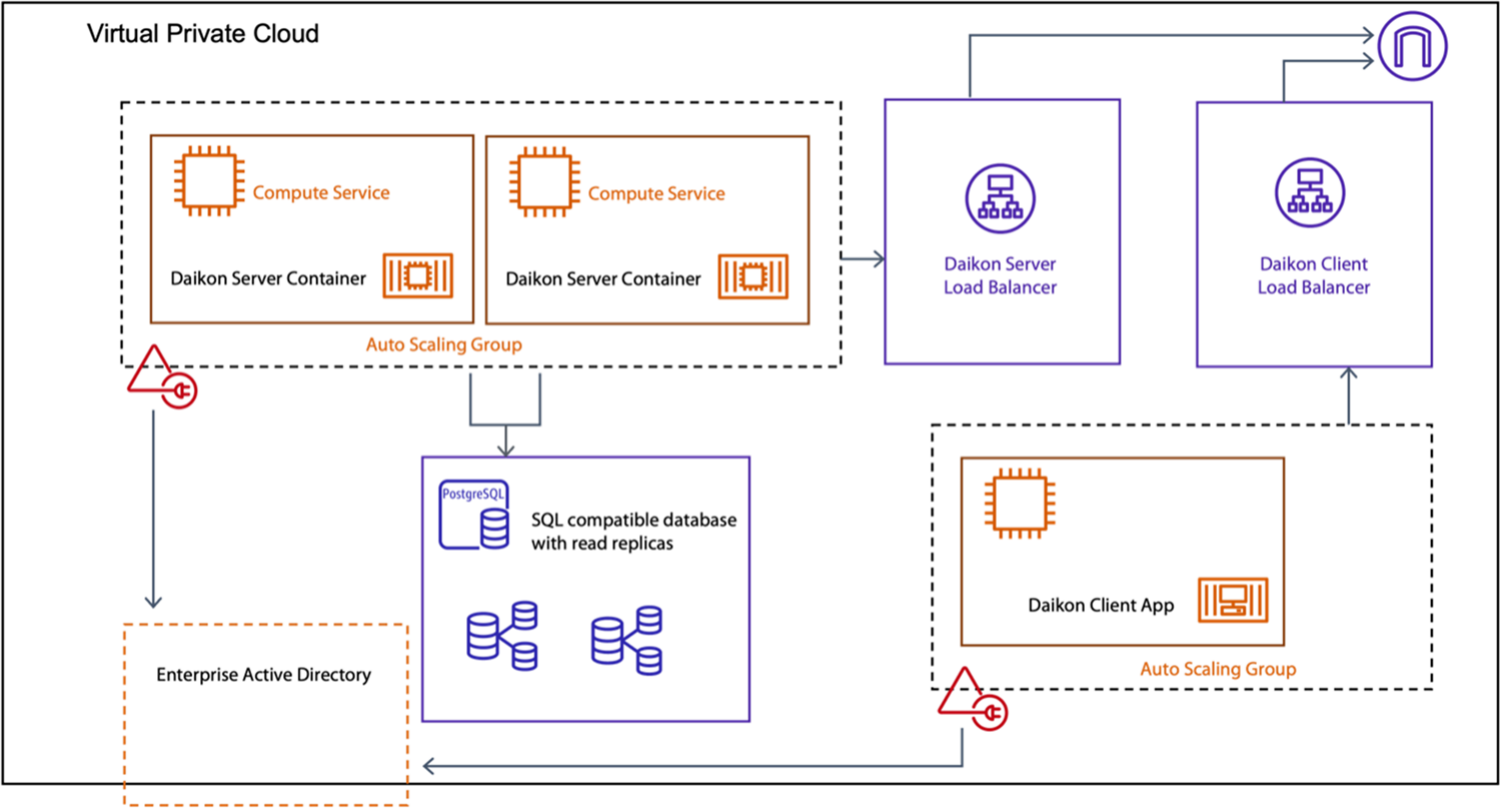
Example deployment for a scaled-up version using dedicated instances for each component, placing them behind load balancers in Amazon Web Services.

We have attempted to simplify deployment processes by utilizing the industry-standard “Platform as a Service”^30^ product “docker” to package and distribute containers that can be provisioned on private/public clouds, Kubernetes clusters, and on-premises by pulling the published images from the hub or by building the application from the source. In addition, docker-compose scripts are provided for smaller groups that prefer to run the suite on a single on-premise server. The application has a layered service-based architecture that can quickly scale from a lab environment to a multi-server deployment for handling a magnitude of requests. Figure 5 demonstrates an example implementation on Amazon Web Service.

## Technology

DAIKON is composed of four core elements: a server-side application to execute business logic with an API interface; a web-based client application to access the data and interact with users; an SQL-compatible database to store the data; and a user authentication bridge to link the organization’s existing Active Directories or SSOs.

### DAIKON Server

**Figure 6:**
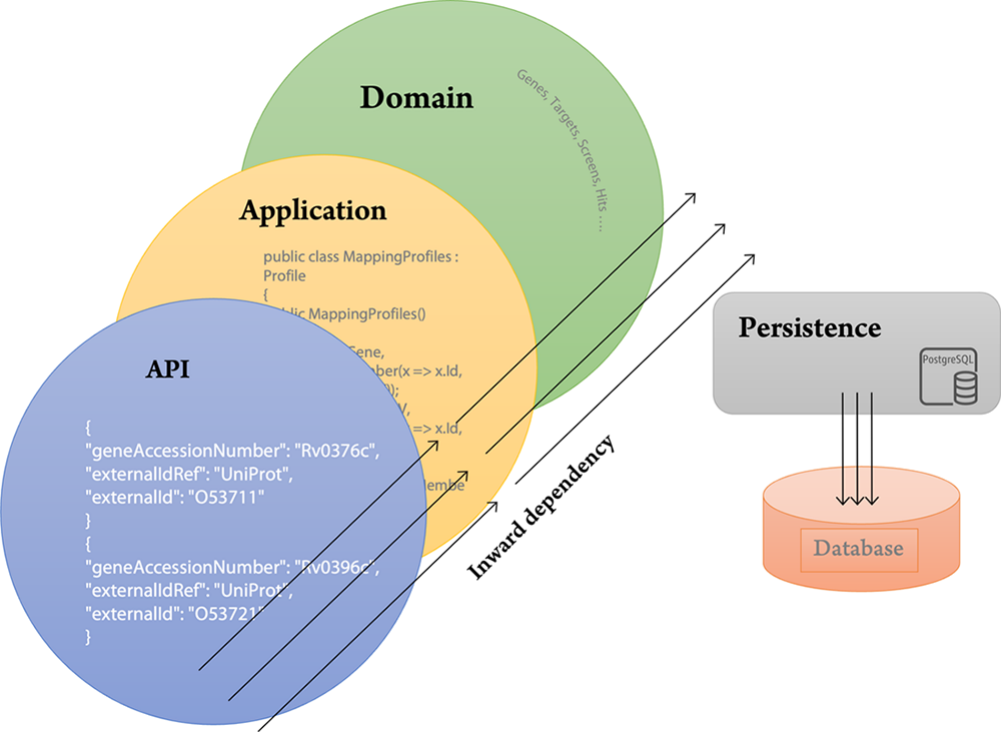
Layered architecture of DAIKON Server.

Due to the complexity and lack of standardization in the drug discovery research protocols, it is expected that business logic will regularly evolve, resulting in a constant flux of design changes to the software architecture, which poses numerous maintenance challenges. A monolithic architecture will necessitate several service rewrites or frequent code refactoring, which will tend to delay deliverables and is therefore not favored in this environment. DAIKON Server is divided into five projects using a layered architecture and a command query responsibility segregation design pattern^31^ to handle this issue. This design partitions read processes (queries) and write operations (commands) into distinct models, making it more maintainable, flexible, and adaptable to modification, particularly in the write compartment, where the more complicated business logic resides. In addition, the layered architecture has a one-way inward dependency, which means that the inner most layer is completely autonomous and oblivious to the presence of layers above it. This permits exterior layers, such as the APIs, to be sufficiently flexible to change without affecting layers underneath them, such as the business logic layer or the database. Figure 6 illustrates the different layers of the DAIKON server app.

The Domain is the innermost layer that defines entities such as genes, targets, etc. that are fundamental to the drug discovery pipeline. This layer establishes domain-specific regulations that model the truth for the drug discovery pipeline’s state and behavior, but it neither stores data nor directly interacts with the database. The application layer encapsulates and is dependent on the Domain layer. This layer contains the implementation of the DAIKON principles and imparts meaning to each entity of the Domain, both individually and collectively. The API is a thin layer that resides over the server’s outermost shell, and its principal objective is to expose the application to the external world using JSON, an open standard file format. In addition, this layer specifies user roles and associates access control policies to authorize and limit access to regulated areas. The persistence layer decouples the DAIKON server from the low-level implementation of underlying relational databases. The infrastructure layer delegates user authentication to directory services such as Azure Active Directory or Keycloak, which guarantees that it is conducted using industry-standard and established secure techniques, also allowing businesses to connect their existing user management processes with the application.

### DAIKON Client

The DAIKON client is a stateless web application built using ReactJS. In addition, various custom components relevant to the discovery pipeline such as the compound evolution timeline and horizon view were developed and may be exported for use in other related projects. The client leverages existing plugins like “SmilesDrawer”^32^ to embed and visualize structures within the application.

## Discussion

Currently, DAIKON is implemented for the TBDA. With the support of the Bill & Melinda Gates Foundation,^33^ TBDA is an innovative partnership of pharmaceutical companies, academia, and research groups working together to understand TB’s pathogenesis better and design high-impact drug candidates. Given the size of many organizations involved and the scale of work undertaken, organizing the data generated, recording results in a centralized data repository, and managing the projects and portfolios were the consortium’s greatest challenges.

In the context of scientific research, the importance of maintaining thorough and organized documentation of screening and hits cannot be overstated. In private industries, strict guidelines and procedures are often put in place to ensure the accurate recording of experimental results. However, in the academic environment, the responsibility for maintaining such records often falls to graduate students, undergraduate students, technicians, and post-doctoral scientists. As a result, research notes were often recorded in a combination of paper and electronic formats, such as laboratory bound notebooks or logbooks, word documents, presentations, and e-notebooks. While there are suggested formats for maintaining these records, they were often dispersed, disorganized, and difficult to interpret. This made it challenging to maintain accurate and up-to-date records of the conditions under which experiments were performed, as well as the quantitative and qualitative results of analyses. When projects are completed or staff members leave the laboratory, the custody of research notes is often transferred, but these notes were not always recorded legibly or organized in a way that facilitated their use. In multidisciplinary collaborations, this information becomes difficult to communicate to the external research community, ultimately hindering the dissemination and use of the knowledge gained. In addition, it is also useful for scientists to view lead molecules categorized by projects and formal HA information and to be able to gain knowledge of target compound inhibitors.

To address these issues, DAIKON provides scientists with a data repository for target selection information, screening hits, and a way of keeping abreast of developments on projects. In a day-to-day setting, lab members regularly and systematically record the results of screening activities, methods, and protocols used in their experiments, dates on which tests were made, validated hits that were obtained, along with structures and concentration in the screen component. Moreover, researchers add notes and comments to provide context and detail about the conduct of their experiments. This allows other skilled scientists to refer to the work and obtain similar results and enables researchers to refer to their original research in the future, if necessary for data analysis, publication, collaboration, peer review, or other research activities. By using the discussion thread feature, scientists collaborate on a common interface and pool their resources to make rapid progress on a given problem. DAIKON allows researchers from diverse disciplines with similar scientific interests to share their findings and communicate with one another globally. This is particularly useful for students, postdoctoral researchers, and chemists who are often spread across the world and may have distinct educational backgrounds.

In program and portfolio management, DAIKON is utilized by managers to analyze the overall status of their discovery portfolio. Visualization of project progress across the pipeline allows for the prediction of milestones and detection of bottlenecks, thus enabling appropriate and effective resource allocation. Participants in meetings, typically comprising program managers, researchers, professors, chemists, and industry collaborators, often use the discussion thread as a means of facilitating open dialogue and teamwork. This not only helps to facilitate a sense of community among the contributors but also serves to drive advancements in their respective fields.

Overall, DAIKON enables users to monitor the status of initiatives aimed at the same target, fostering collaboration, and minimizing the likelihood of duplication of effort.

The modular framework was initially designed for *Mycobacterium tuberculosis*, but it is not restricted to TB genes. Users can import genes for other organisms by submitting a JSON document containing an array of gene data through an API call to the DAIKON Server. For TB genes, the majority of the organism and strain data can be obtained from Mycobrowser, and DAIKON includes an “adapter” that automatically extracts, transforms, and loads data from Mycobrowser for all the organisms it encompasses. A similar “adapter” for UniProt is being developed that is intended to cover a broader range of organisms and can be utilized to query UniProt through searches and filters, enabling users to access and import gene data directly from Uniprot to DAIKON. In addition, we are actively developing this framework to embrace the specialized customization of discovery pipelines for SARS-CoV-2 and parasitic diseases such as malaria to provide out-of-the-box standardized templates. We are also working toward deeper integration of the workflow with molecular databases,^34^ in-app processing tools for compound clustering, and other project management features to make the implementation much more straightforward.

In conclusion, DAIKON is a cloud-based web application that integrates multiple source systems across many data points to create a unified platform for drug discovery research. Information is shared seamlessly between research members and managers, and effective end-to-end data tracking provides aid to discovery research and pre-clinical trials.

The framework is freely available at https://github.com/saclab/daikon-core-server, https://github.com/saclab/daikon-core-webapp

## Supporting Information Available

Screenshots from DAIKON, intended to aid in understanding the application’s functionality, including those of the DAIKON’s user interface, examples of a gene in a specific stage of the pipeline, and horizon views and compound evolutions generated by DAIKON (PDF)

## Abbreviations

API: Application Programming Interface
CQRS: Command and Query Responsibility Segregation
CRUD: Create Read Update and Delete
GUI: Graphical User Interface
H2L: Hit-to-Lead
HA: Hit Assessment
IC50: Inhibitory Concentration 50
IND: Investigational New Drug
JSON: JavaScript Object Notation
LO: Lead Optimization
MIC: Minimum Inhibitory Concentration
PDB: Protein Data Bank
SQL: Structured Query Language
SSO: Single Sign On
TB: Tuberculosis
TBDA: TB Drug Accelerator program
VPC: Virtual Private Cloud
