# Supplementary material for: DAIKON: A Data Acquisition, Integration, and Knowledge Capture Web Application for Target-Based Drug Discovery
Source: ACS Pharmacol Transl Sci. 2023 Jun 22;6(7):1043–51. doi: 10.1021/acsptsci.3c00034 (PMC10353056; doi:10.1021/acsptsci.3c00034)
Supplement: Supplementary file 1 — pt3c00034_si_001.pdf [file pt3c00034_si_001.pdf]

SUPPORTING INFORMATION

DAIKON: A Data acquisition, integration, and knowledge capture web application for target-based drug discovery

Siddhant Rath[1], Saswati Panda[1], James C. Sacchettini[1], Steven J. Berthel[1,2].*

[1] Department of Biochemistry & Biophysics, Texas A&M University, College Station, TX 77843

[2] Panorama Global, Seattle WA 98121

AUTHOR INFORMATION

Corresponding Author

James C. Sacchettini, Ph.D.
Professor of Biochemistry and Biophysics
Wolfe-Welch Chair in Science,
Texas A&M University
979-862-7637
sacchett@tamu.edu

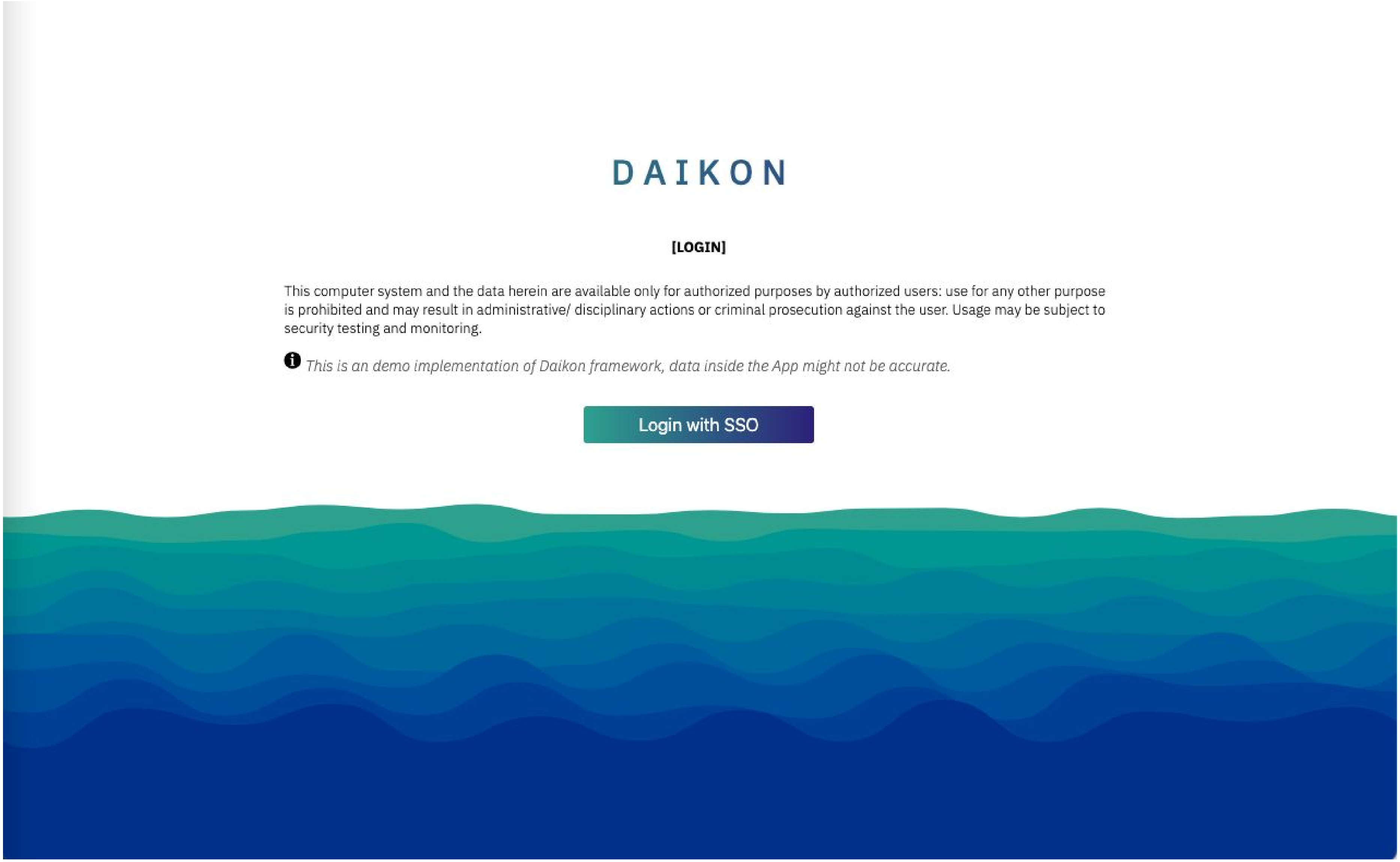

Figure S1 Login Screen.

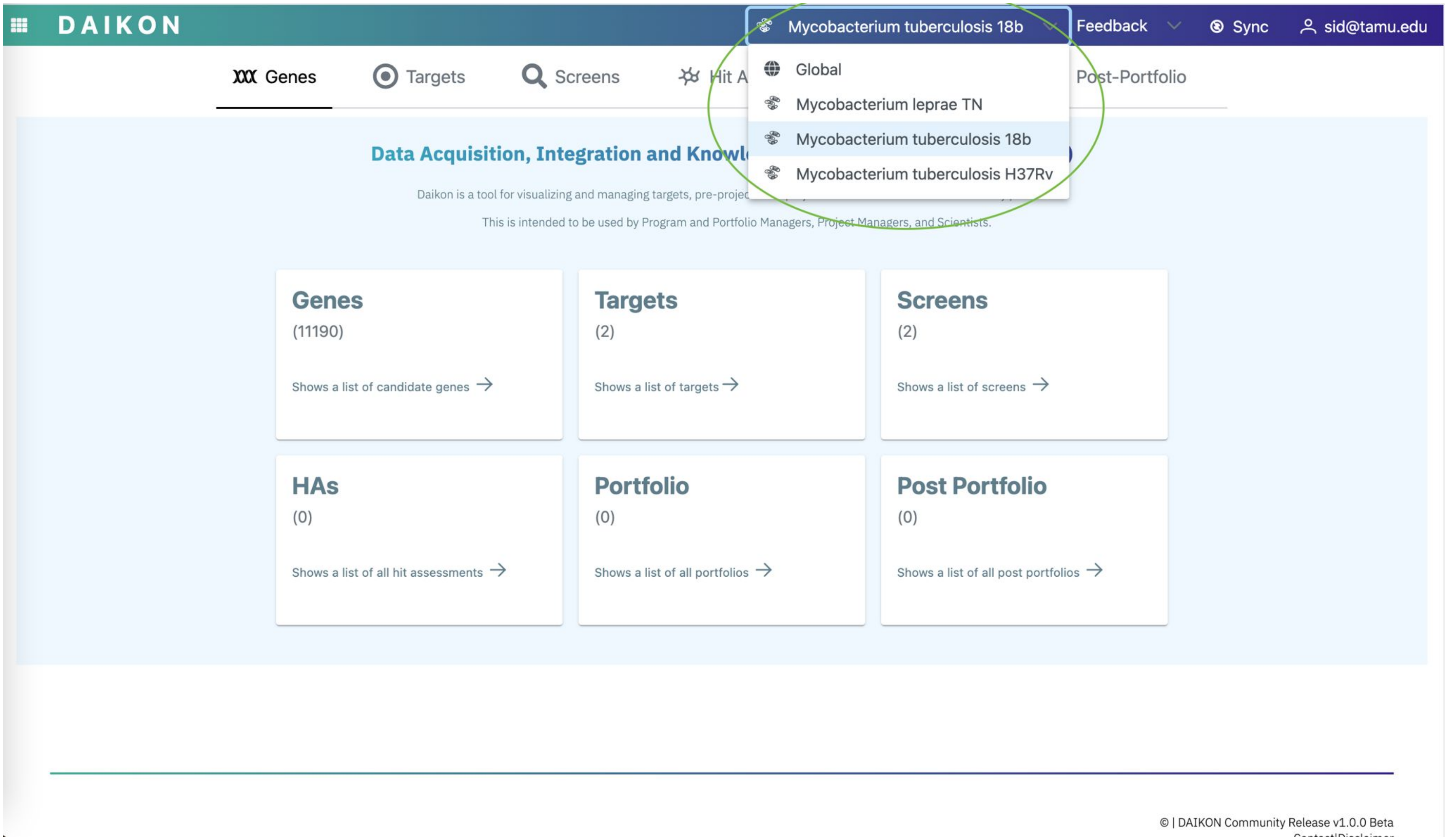

Figure S2 Organism / Strain Filter.

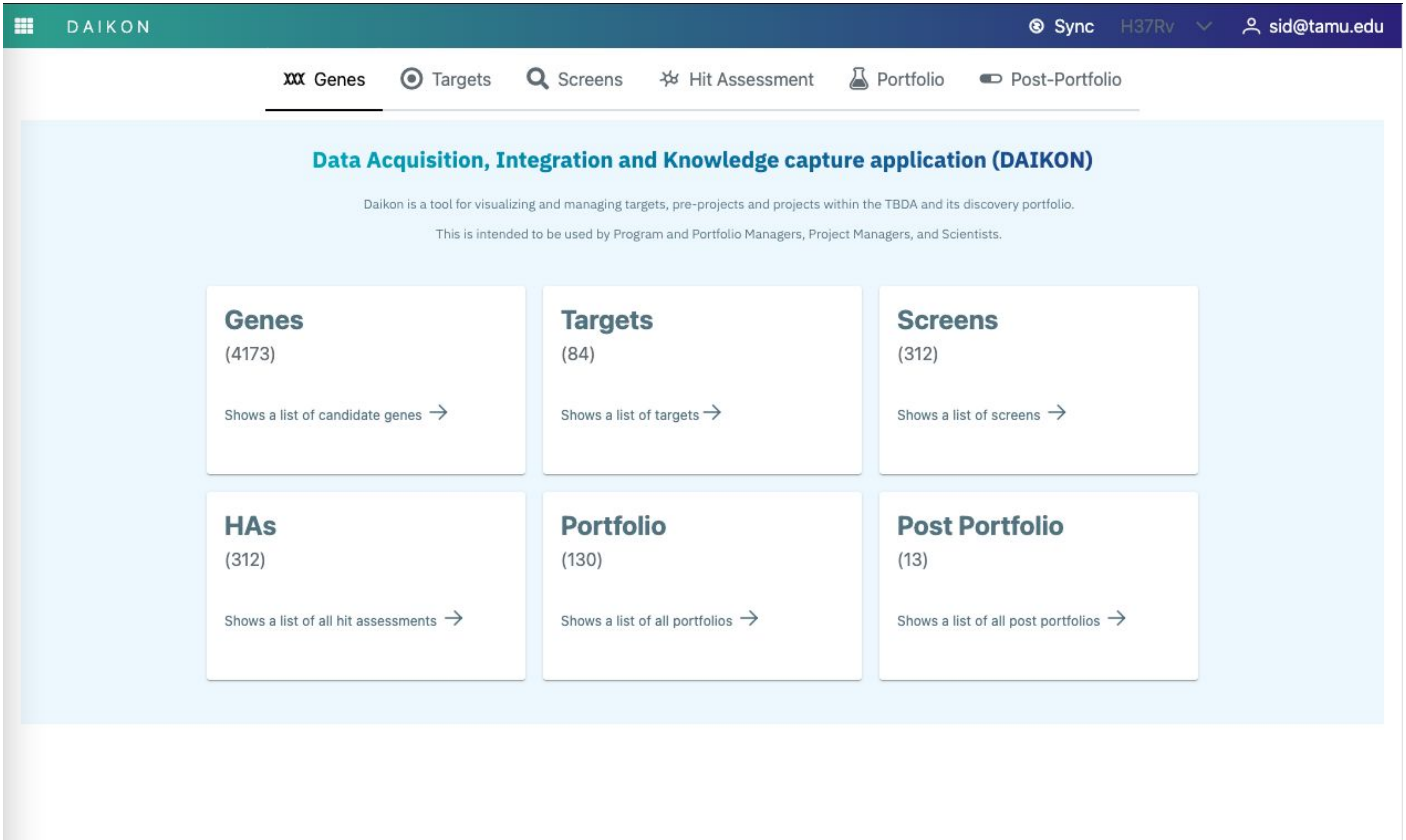

Figure S3 Landing Dashboard, displaying the number of entries in each stage.

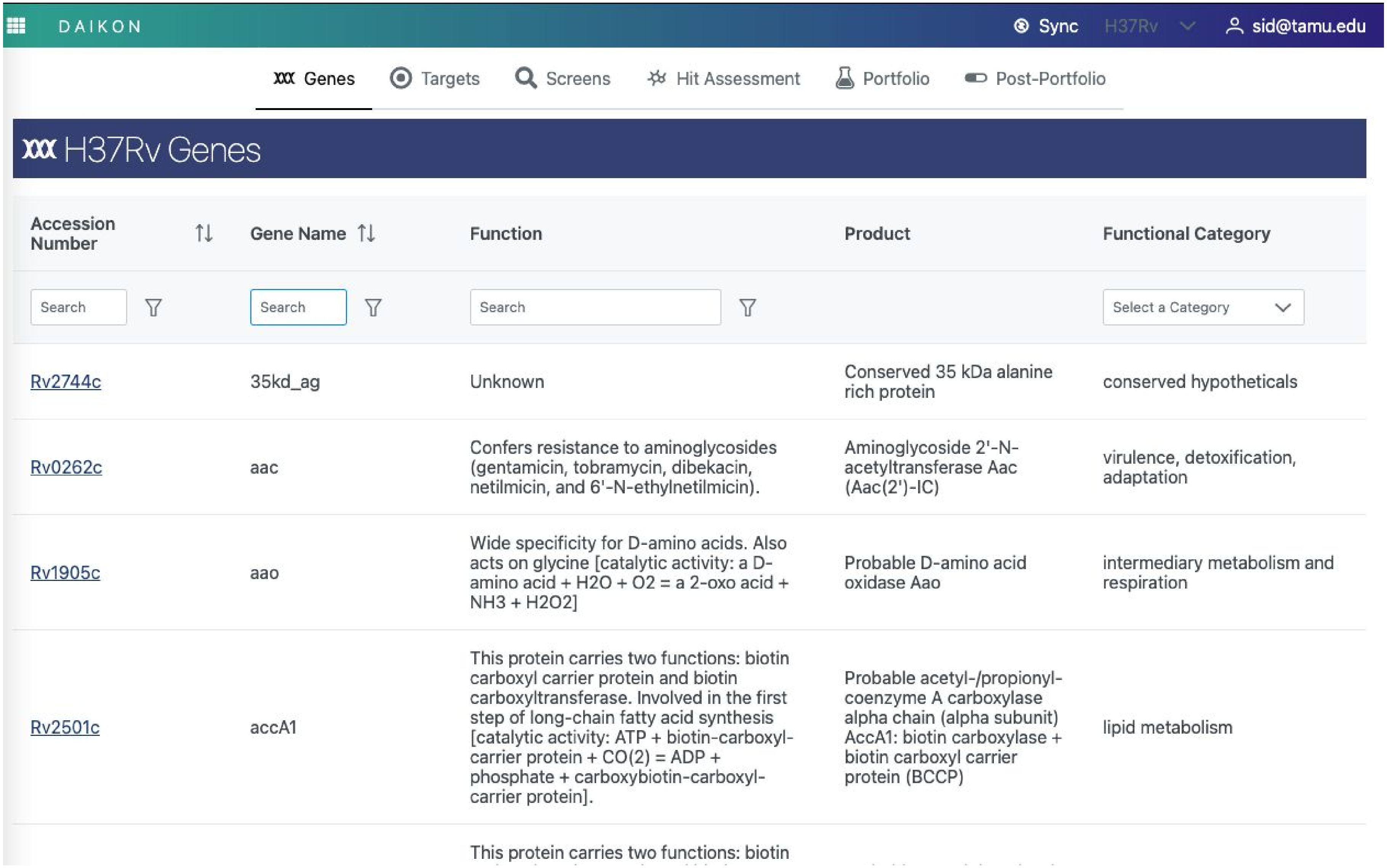

Figure S4 Gene Component listing all the Genes of the selected organism/strain filter (in this case H37Rv).

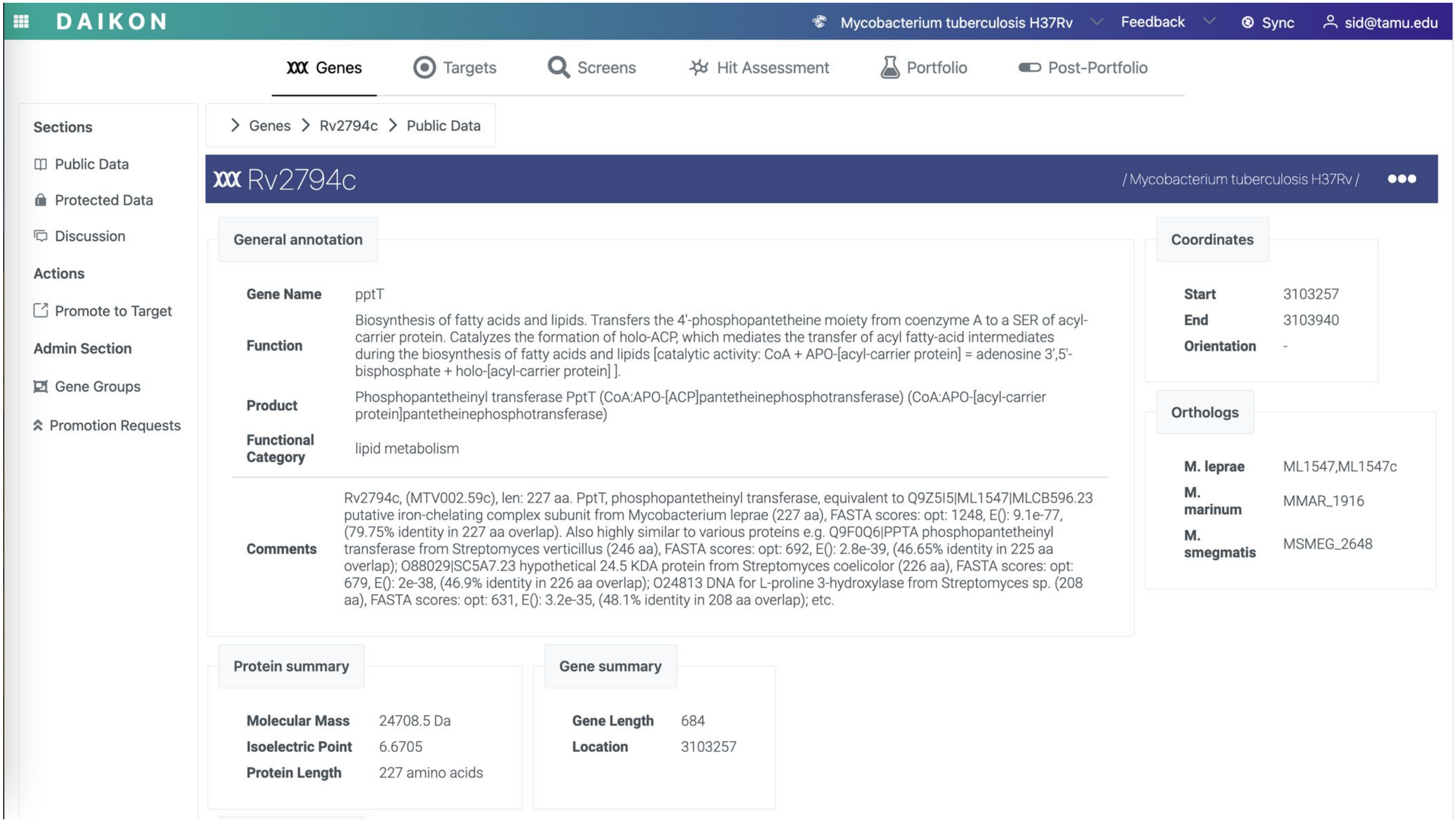

Figure S5 Further Details for each gene.

Protein summary

**Molecular Mass**	24708.5 Da
**Isoelectric Point**	6.6705
**Protein Length**	227 amino acids

Gene summary

**Gene Length**	684
**Location**	3103257

Protein databank

PDP ID	Method	Resolution	Chains	Ligands	Structure
7N8E	X-ray	1.74 A	A/B=1-227	2 x COENZYME A<br>6 x MAGNESIUM ION<br>2 x N-[2,6-di(propan-2-yl)phenyl]-N'-(N-ethylcarbamimidoyl)urea<br>1 x DIMETHYL SULFOXIDE	View Structure
4QJK	X-ray	1.59 A	A=1-227	1 x COENZYME A<br>2 x SULFATE ION	View Structure
4QVH	X-ray	1.75 A	A/B=1-227	2 x COENZYME A<br>2 x MAGNESIUM ION<br>7 x GLYCEROL<br>2 x CITRATE ANION<br>4 x alpha-D-glucopyranose	View Structure
6CT5	X-ray	1.76 A	A/B=1-227	2 x COENZYME A<br>3 x N-(2,6-diethylphenyl)-N'-(N-ethylcarbamimidoyl)urea<br>6 x MAGNESIUM ION<br>6 x GLYCEROL<br>7 x DIMETHYL SULFOXIDE	View Structure
4U89	X-ray	1.40 A	A=1-227	1 x COENZYME A<br>11 x SODIUM ION<br>1 x PHOSPHATE ION<br>1 x TETRAETHYLENE GLYCOL<br>1 x IMIDAZOLE	View Structure
7N8L	X-ray	2.26 A	A/B=1-227	2 x COENZYME A<br>2 x N-(2,6-diethylphenyl)-N'-(N-propylcarbamimidoyl)urea<br>4 x MAGNESIUM ION<br>3 x GLYCEROL<br>1 x DIMETHYL SULFOXIDE	View Structure

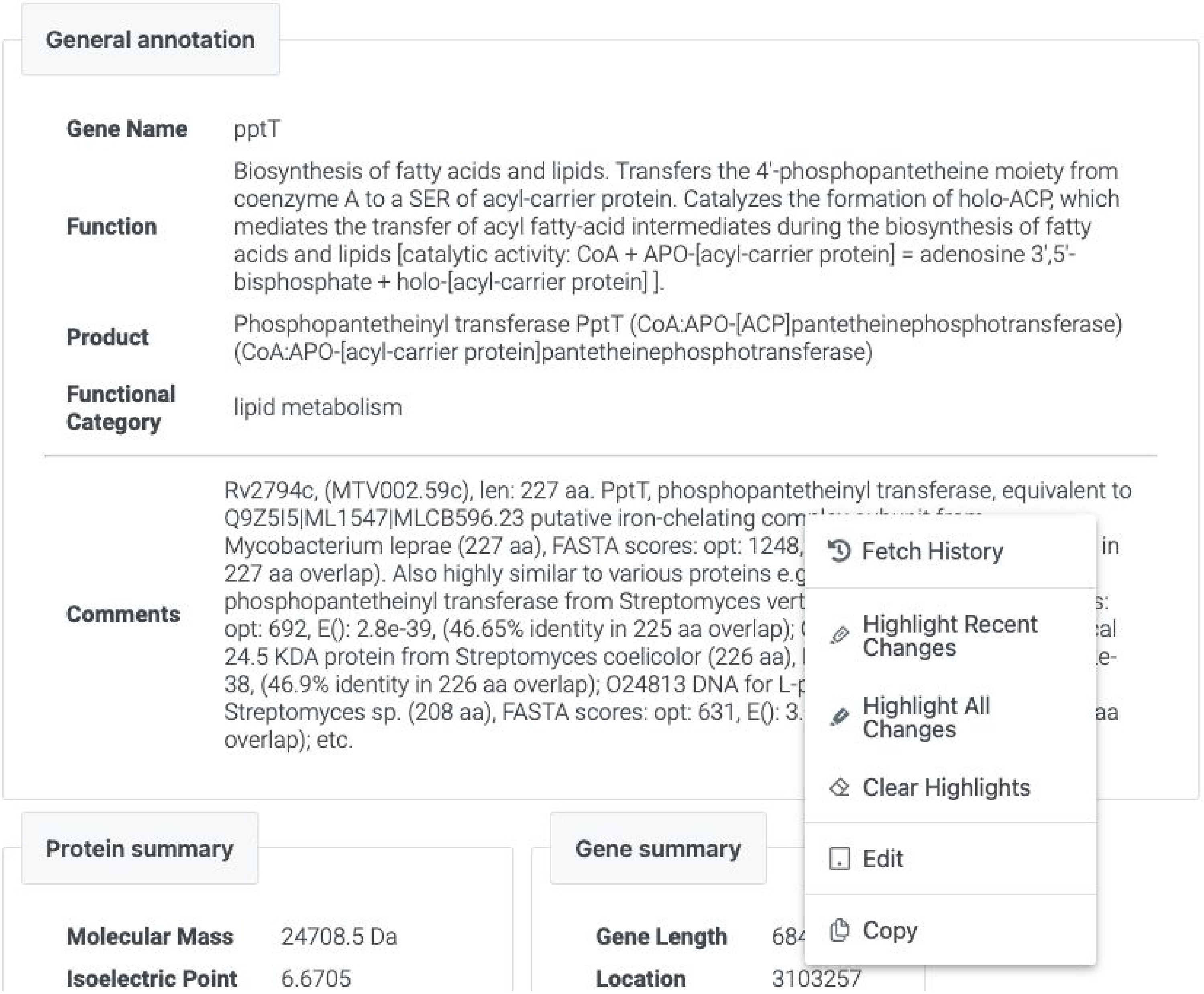

Figure S6 Version Tracking via Fetch History/Highlight Changes. The app can be right clicked at any field to pull a context menu.

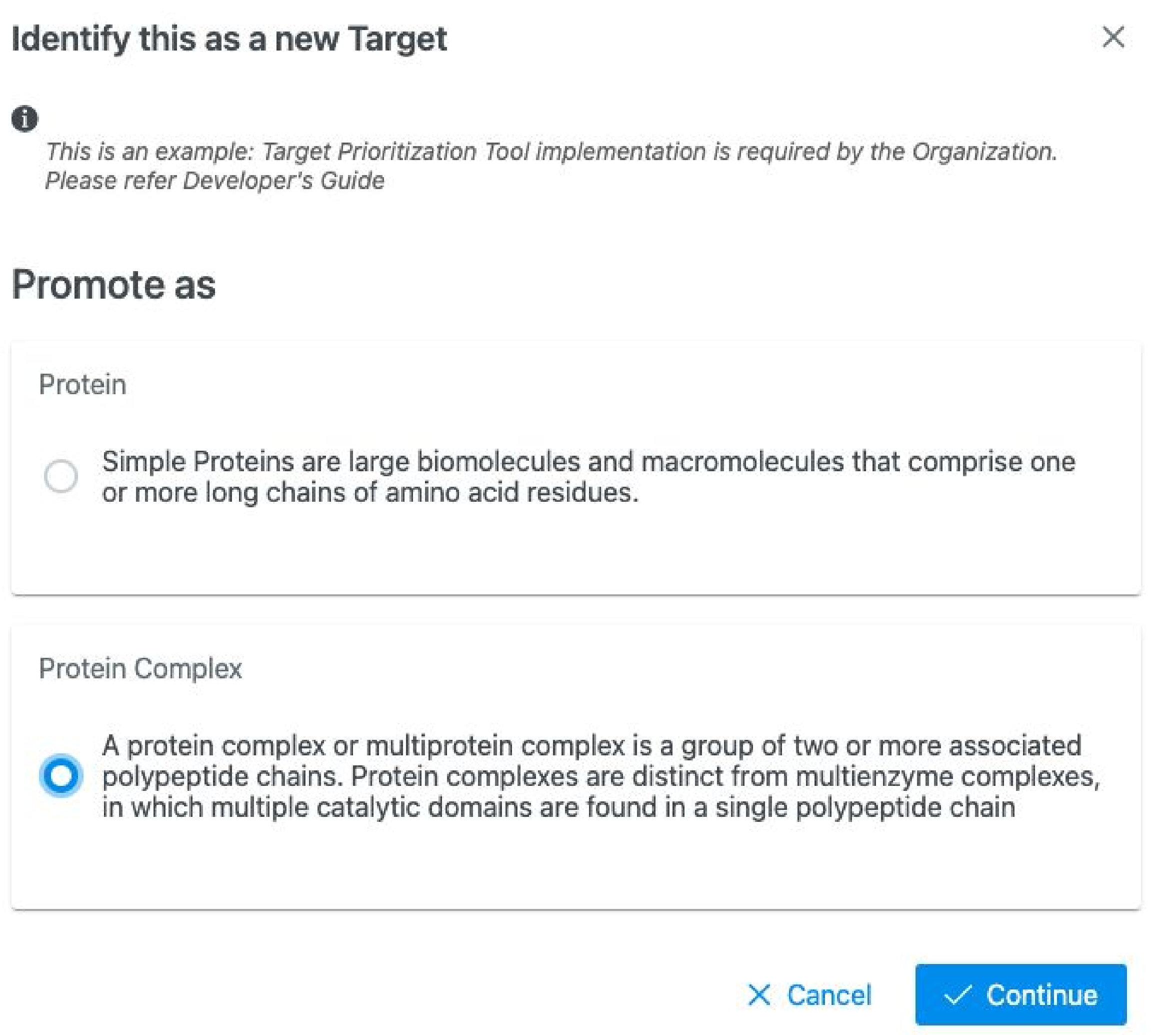

Figure S7 Progressing a gene to a target via the Target Prioritization Module.

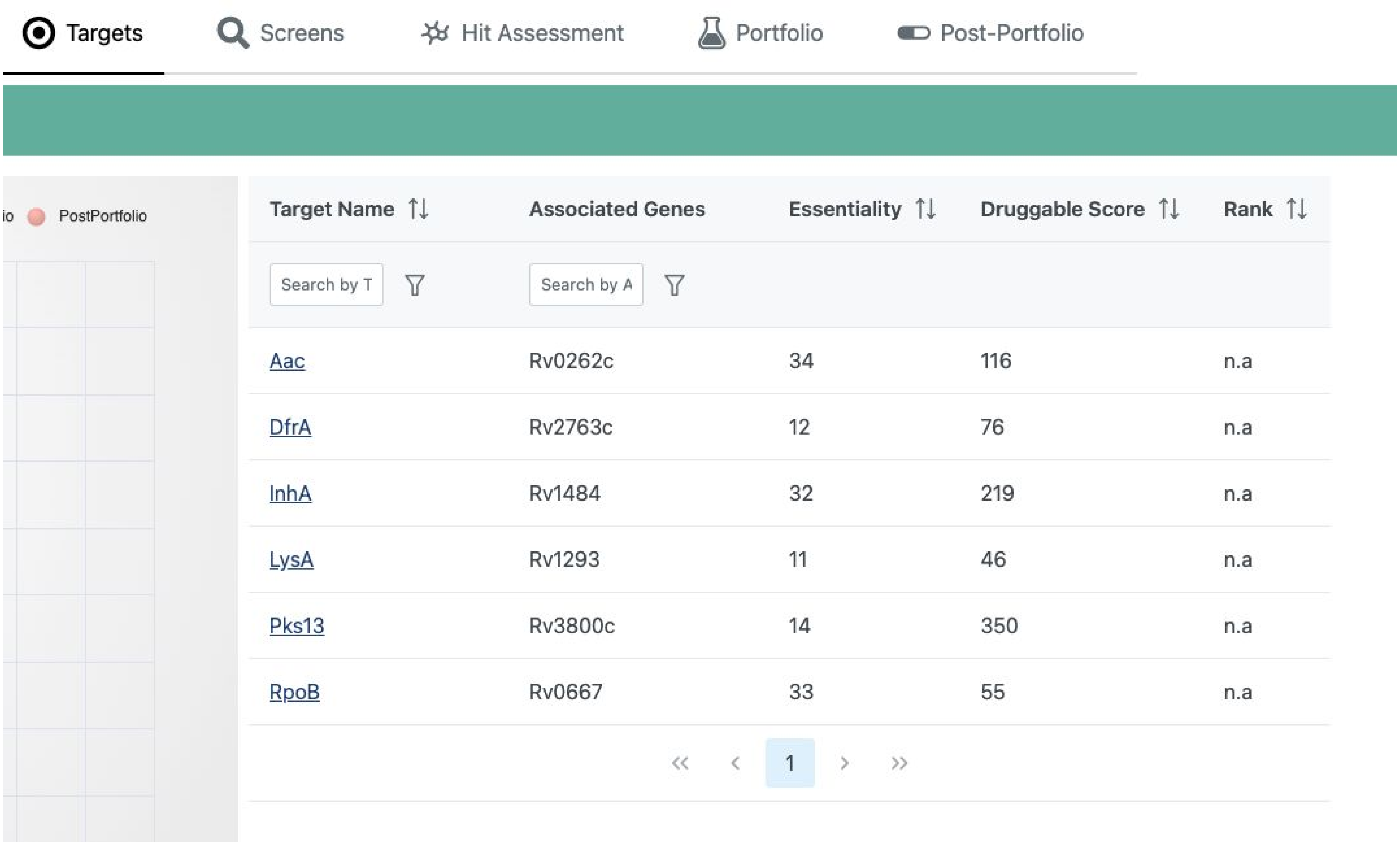

Target Name	Associated Genes	Essentiality	Druggable Score	Rank
Aac	Rv0262c	34	116	n.a
DfrA	Rv2763c	12	76	n.a
InhA	Rv1484	32	219	n.a
LysA	Rv1293	11	46	n.a
Pks13	Rv3800c	14	350	n.a
RpoB	Rv0667	33	55	n.a

Figure S8 Target Component listing the targets.

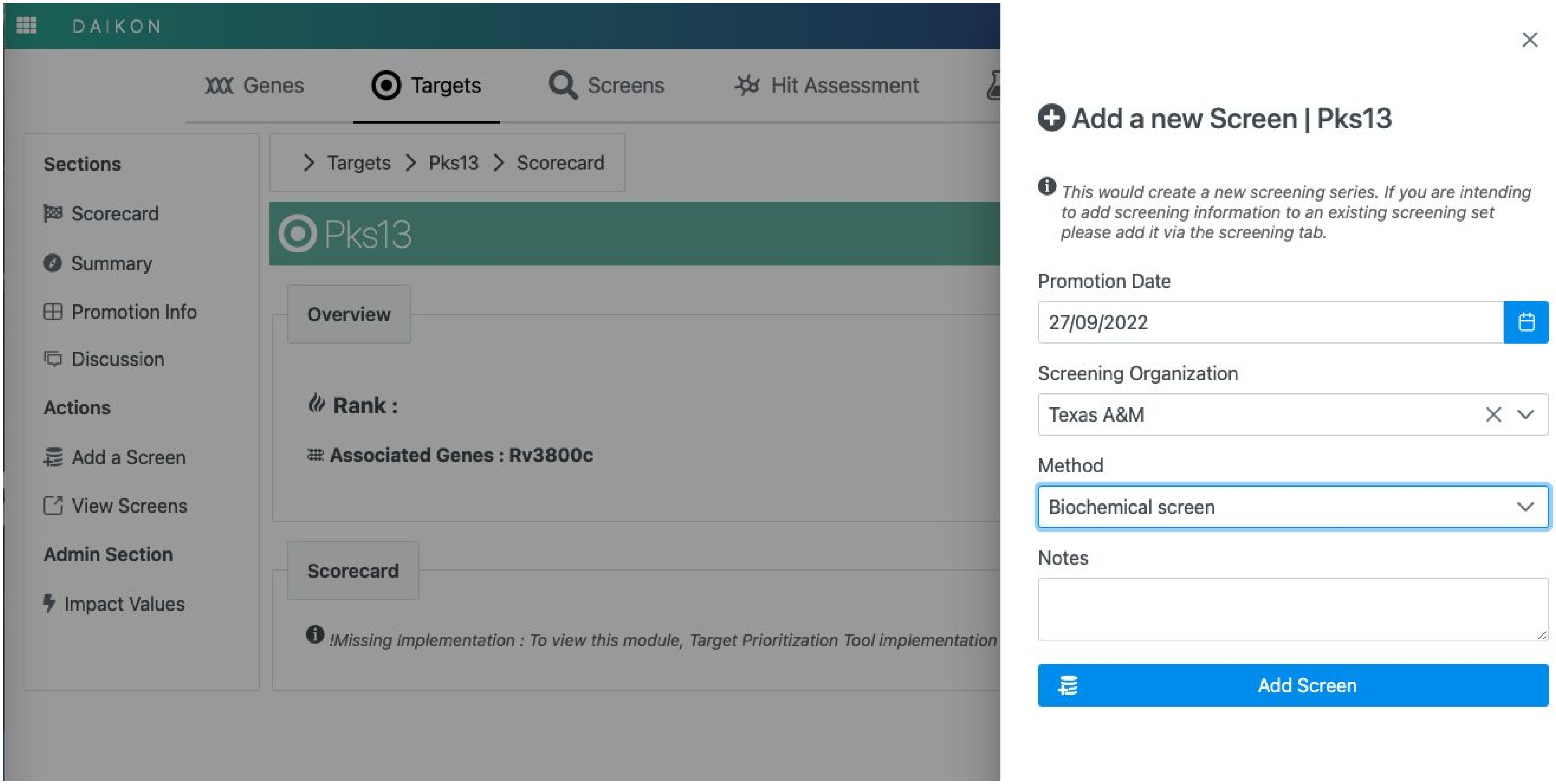

Figure S9 Adding a screen run for a target.

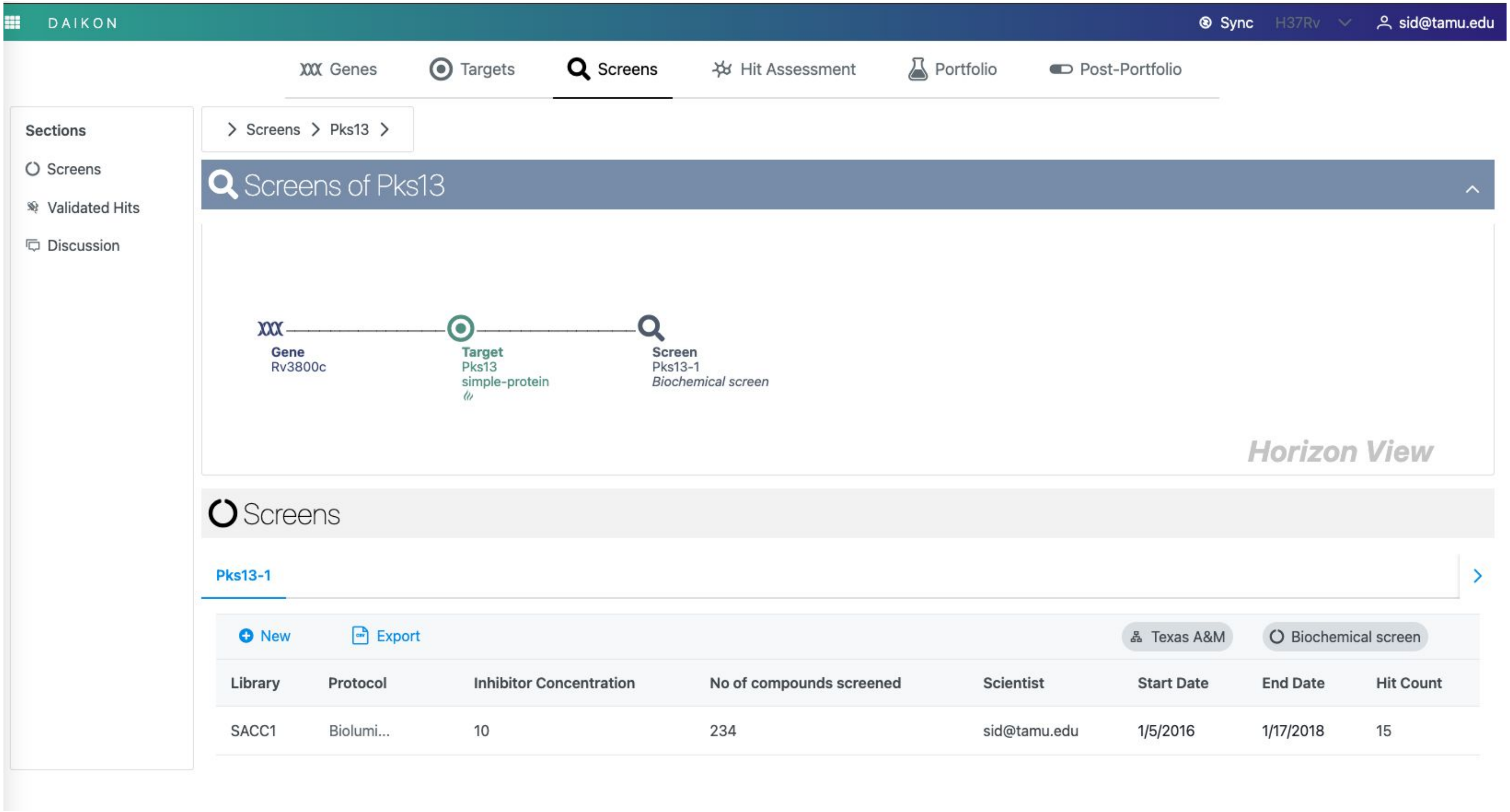

Figure S10 Screen Component showcasing Horizon View and screen runs.

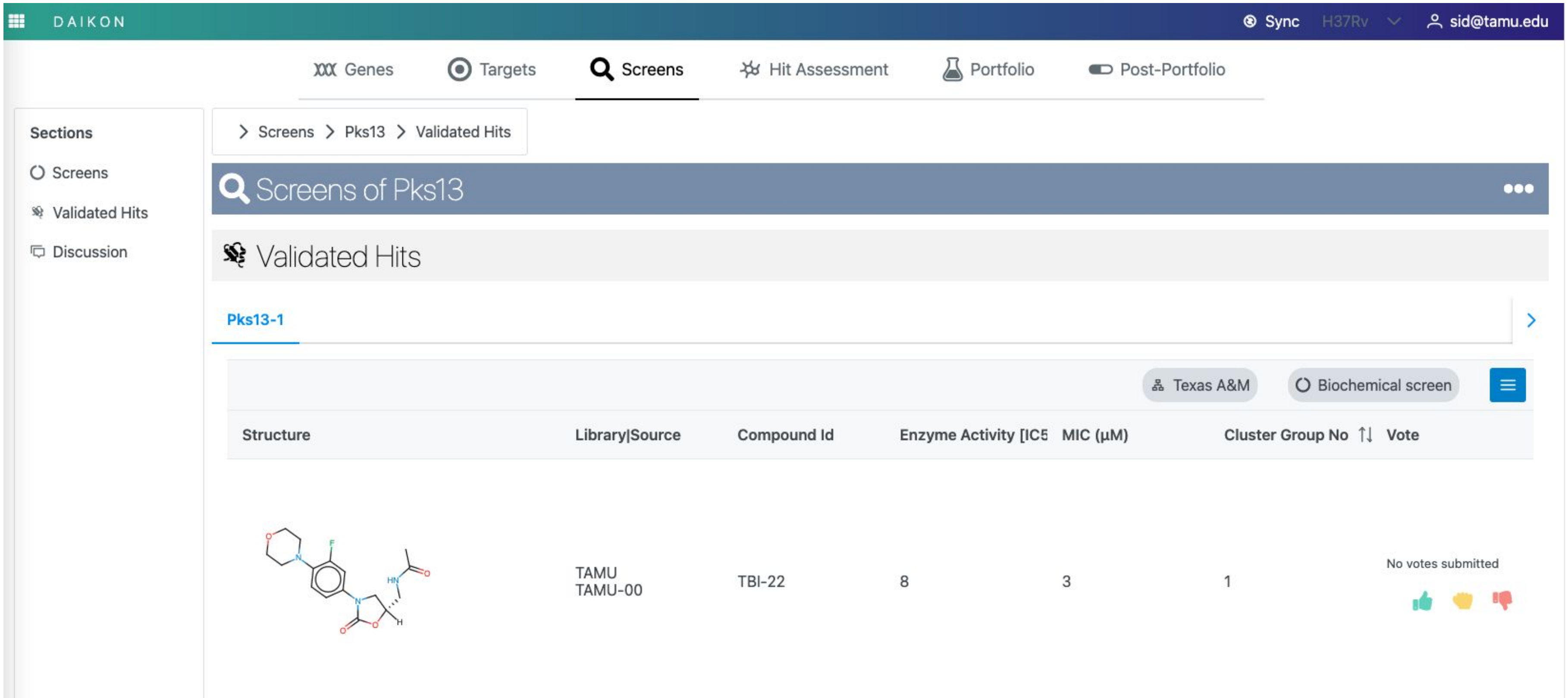

Figure S11 Screen Component listing validated hits with voting.

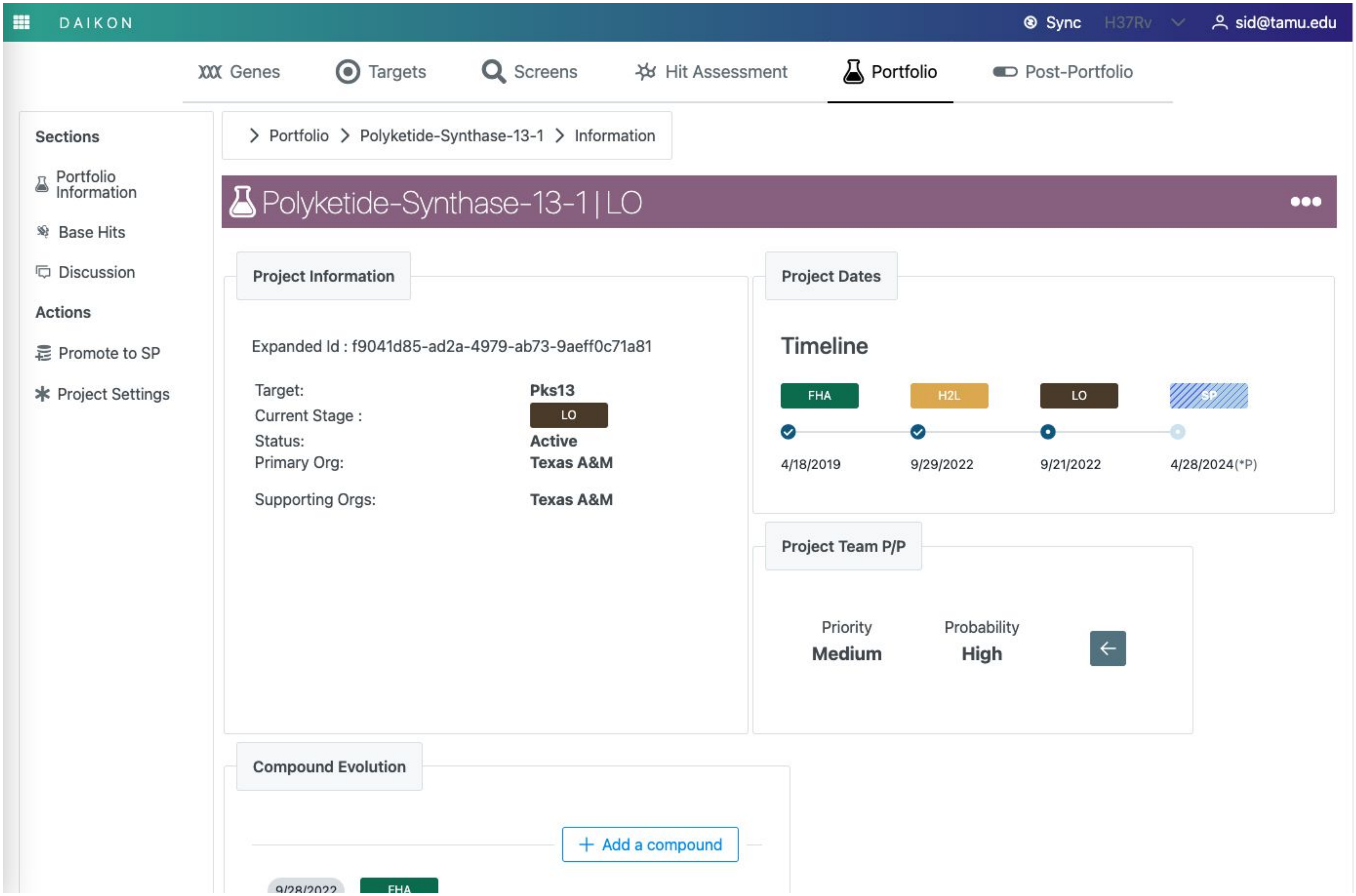

Figure S12 Portfolio Component.

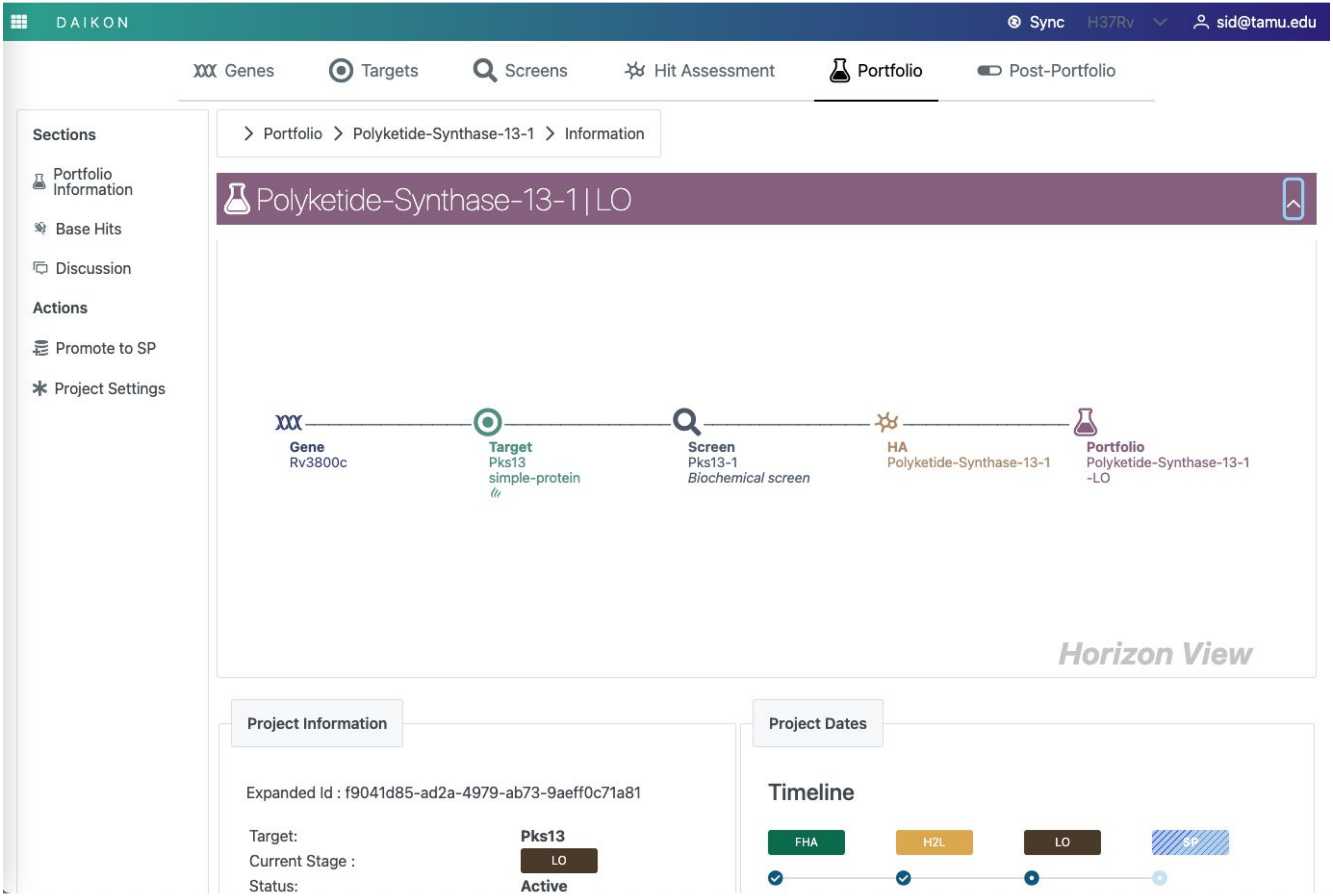

Figure S13 Horizon view spawned in Portfolio Component.

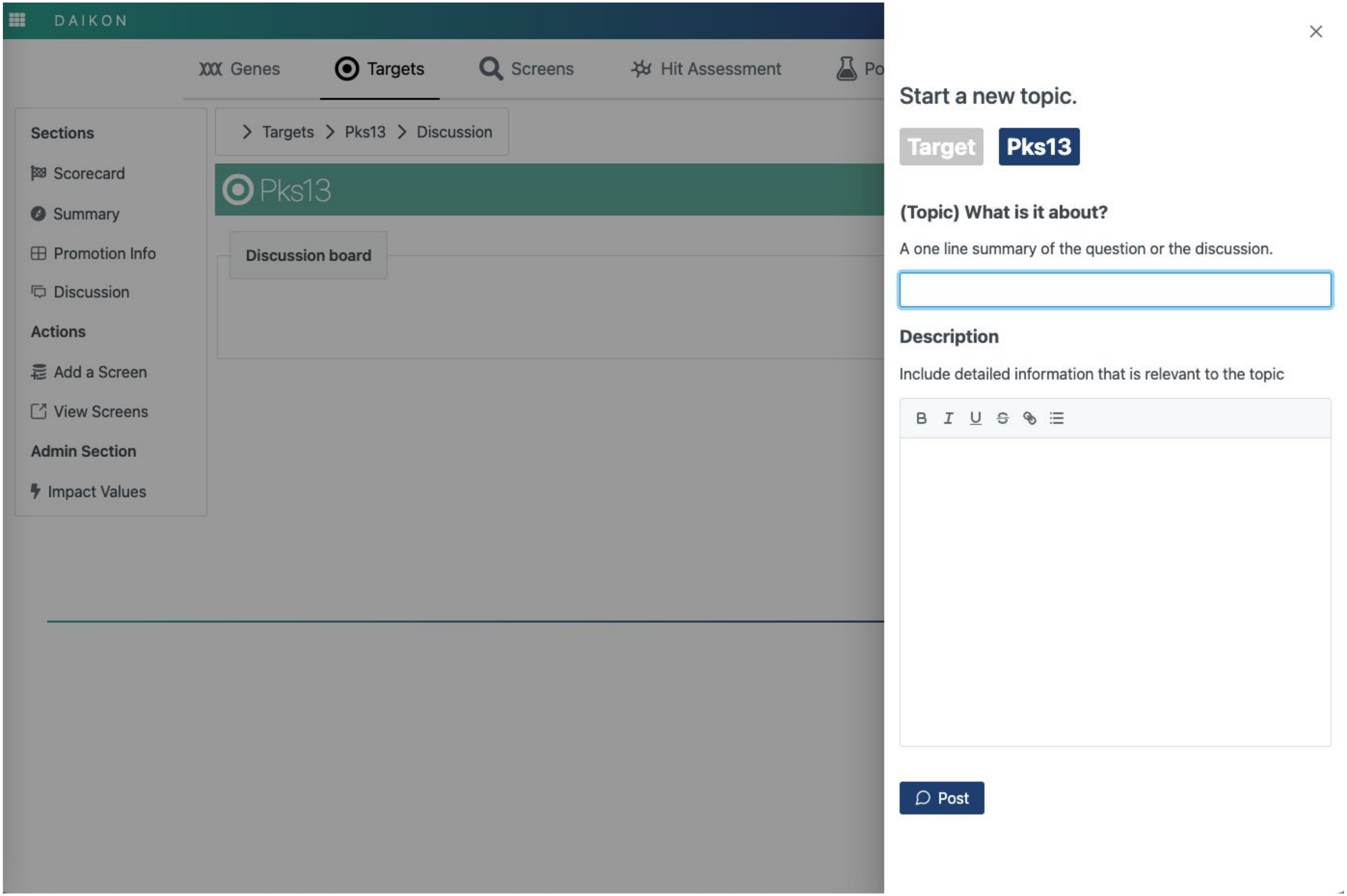

Figure S14 Discussion Thread.

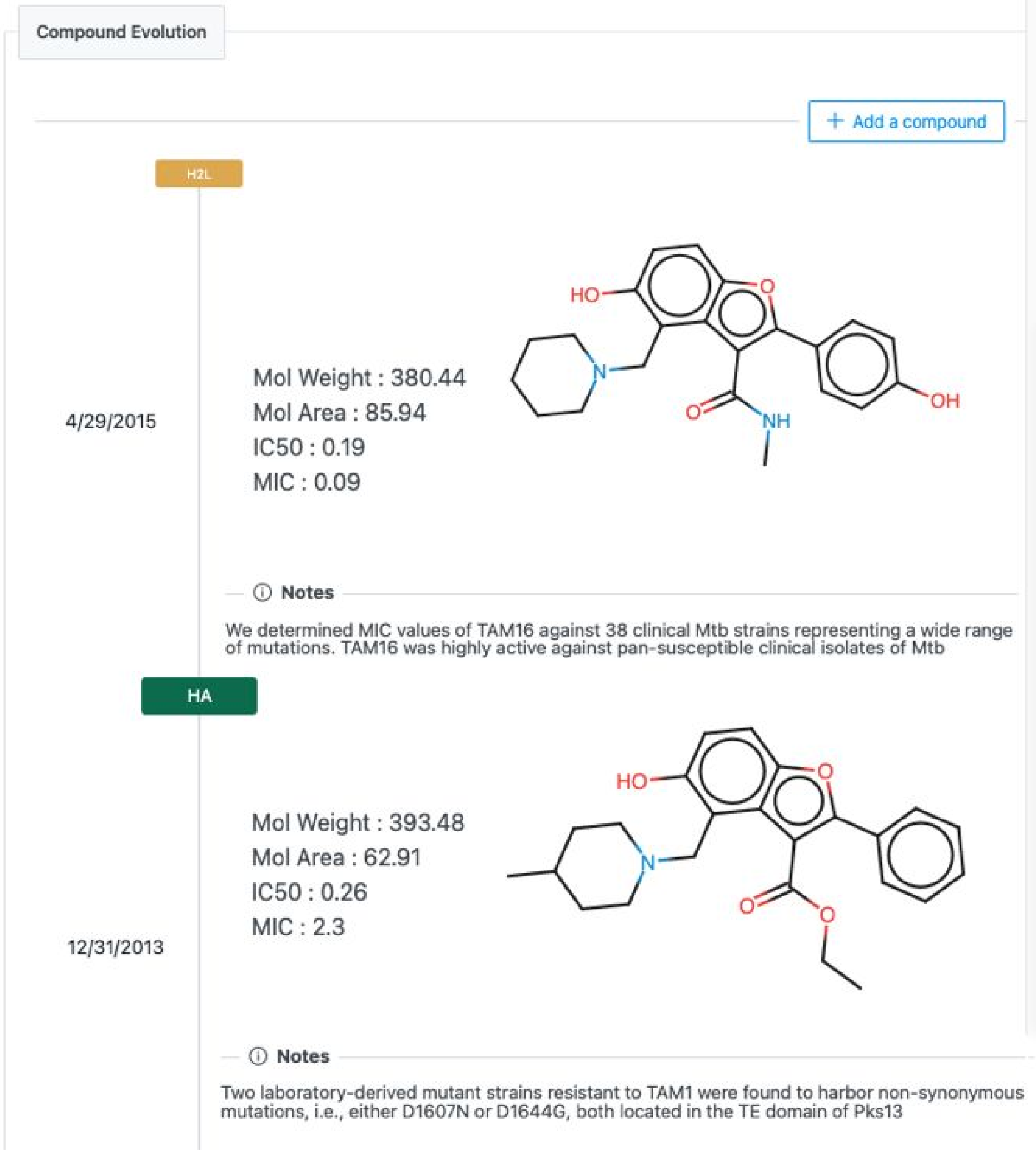

Figure S15 Compound Evolution Component.

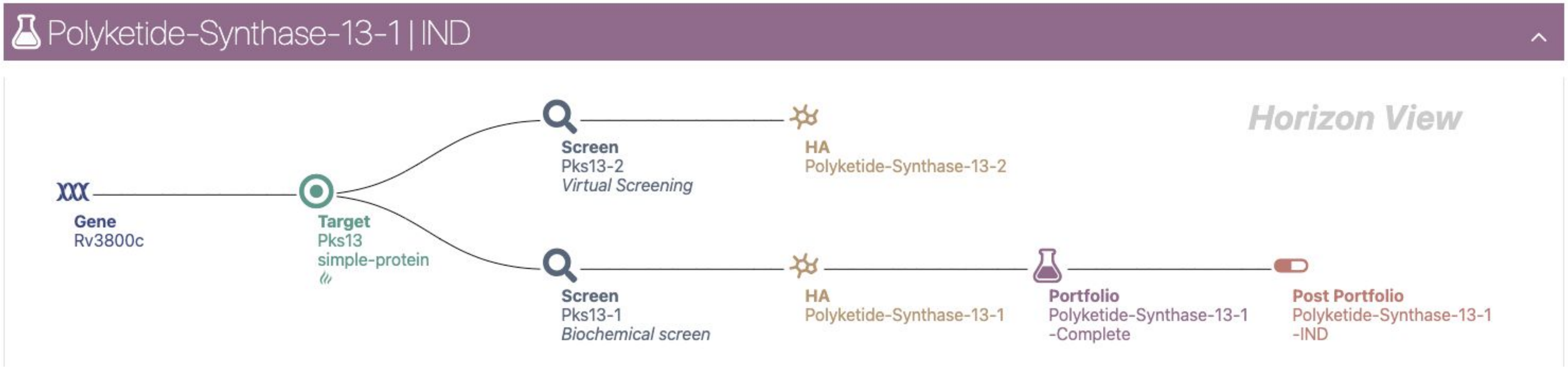

Figure S16 Horizon view capturing multiple journeys of Pks13.

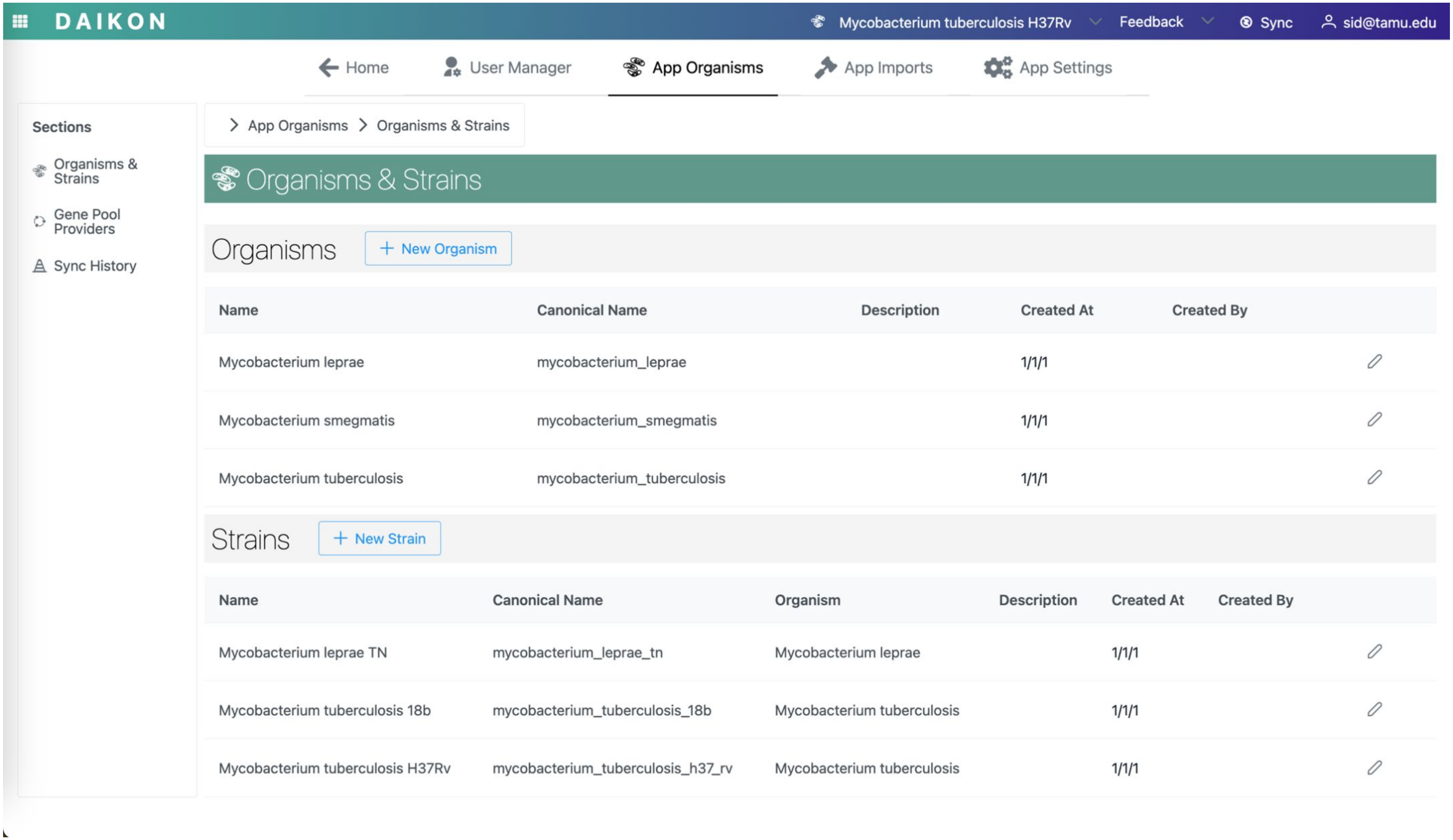

Figure S17 Admin action to handle organisms and strains.

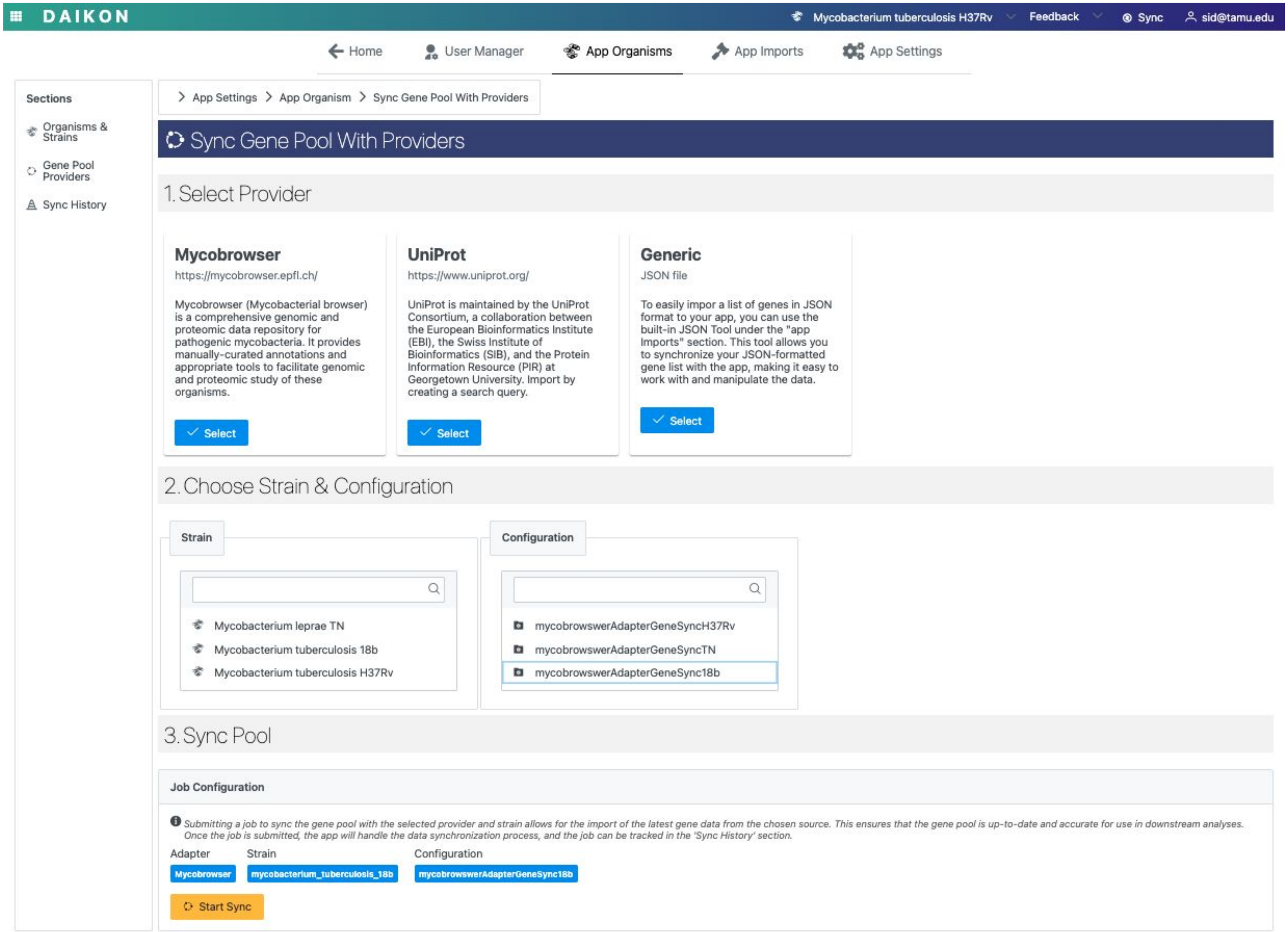

Figure S18 Inbuilt Adapters to import genes from Mycobrowser and UniProt.

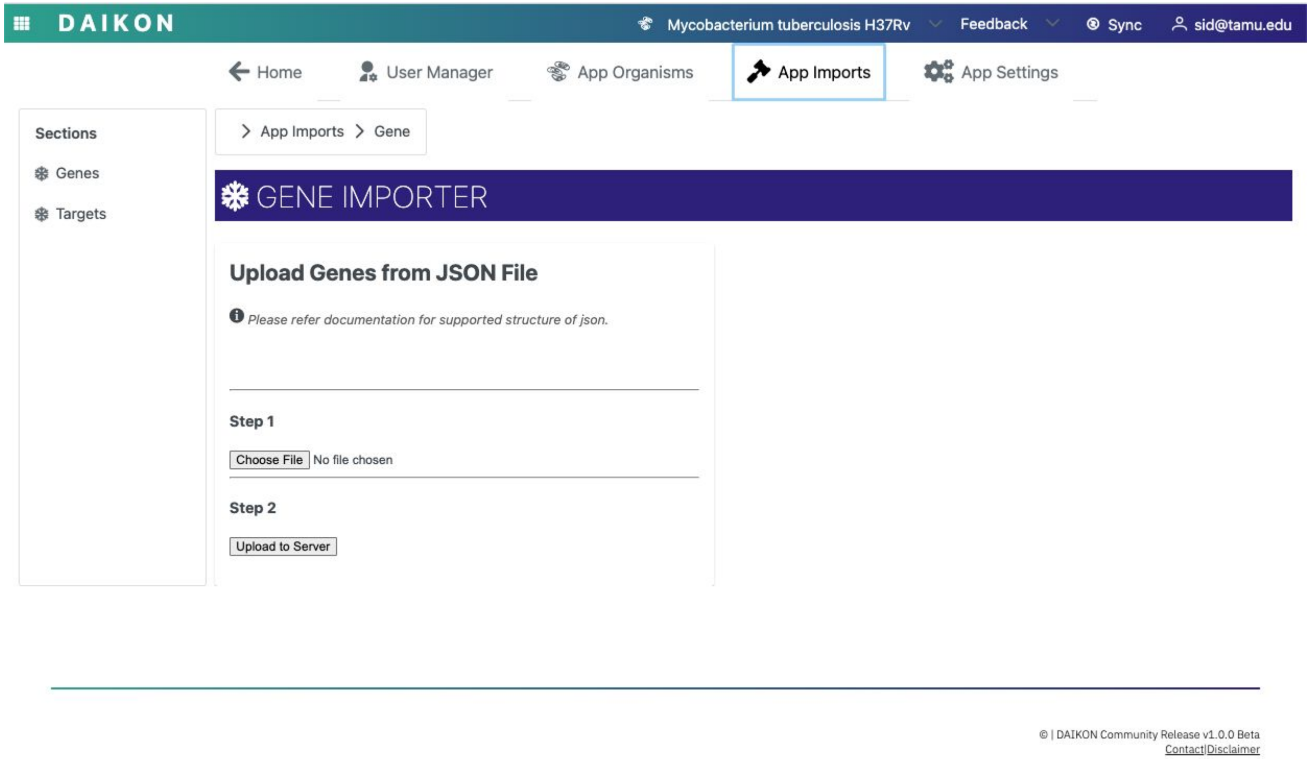

Figure S19 Admin feature to import genes to the app from a JSON file using the generic adapter.
